# Chromatin Organization Governs Transcriptional Response and Plasticity of Cancer Stem Cells

**DOI:** 10.1002/advs.202407426

**Published:** 2025-03-07

**Authors:** Yinu Wang, Jane Frederick, Karla Isabel Medina, Elizabeth Thomas Bartom, Luay Matthew Almassalha, Yaqi Zhang, Greta Wodarcyk, Hao Huang, I Chae Ye, Ruyi Gong, Cody Levi Dunton, Alex Duval, Paola Carrillo Gonzalez, Joshua Pritchard, John Carinato, Iuliia Topchu, Junzui Li, Zhe Ji, Mazhar Adli, Vadim Backman, Daniela Matei

**Affiliations:** ^1^ Department of Obstetrics and Gynecology Northwestern University Chicago IL 60611 USA; ^2^ Department of Biomedical Engineering Northwestern University Evanston IL 60208 USA; ^3^ Center for Physical Genomics and Engineering Northwestern University Evanston IL 60208 USA; ^4^ Chemistry of Life Processes Institute Northwestern University Evanston IL 60208 USA; ^5^ Department of Biochemistry and Molecular Genetics Northwestern University Chicago IL 60611 USA; ^6^ Department of Preventive Medicine Northwestern University Chicago IL 60611 USA; ^7^ Robert H. Lurie Comprehensive Cancer Center Northwestern University Chicago IL 60611 USA; ^8^ Department of Gastroenterology and Hepatology Feinberg School of Medicine Chicago IL 60611 USA; ^9^ Department of Pharmacology Feinberg School of Medicine Chicago IL 60611 USA; ^10^ Department of Pharmacology Department of Biomedical Engineering McCormick School of Engineering Northwestern University Evanston IL 60208 USA; ^11^ Jesse Brown Veterans’ Affairs Medical Center Chicago IL 60612 USA

**Keywords:** cancer stem cells, cell plasticity, chromatin organization, ovarian cancer, transcriptional heterogeneity

## Abstract

Chromatin organization regulates transcription to influence cellular plasticity and cell fate. We explored whether chromatin nanoscale packing domains are involved in stemness and response to chemotherapy. Using an optical spectroscopic nanosensing technology we show that ovarian cancer‐derived cancer stem cells (CSCs) display upregulation of nanoscale chromatin packing domains compared to non‐CSCs. Cleavage under targets and tagmentation (CUT&Tag) sequencing with antibodies for repressive H3K27me3 and active H3K4me3 and H3K27ac marks mapped chromatin regions associated with differentially expressed genes. More poised genes marked by both H3K4me3 and H3K27me3 were identified in CSCs vs. non‐CSCs, supporting increased transcriptional plasticity of CSCs. Pathways related to Wnt signaling and cytokine‐cytokine receptor interaction were repressed in non‐CSCs, while retinol metabolism and antioxidant response were activated in CSCs. Comparative transcriptomic analyses showed higher intercellular transcriptional heterogeneity at baseline in CSCs. In response to cisplatin, genes with low baseline expression levels underwent the highest upregulation in CSCs, demonstrating transcriptional plasticity under stress. Epigenome targeting drugs downregulated chromatin packing domains and promoted cellular differentiation. A disruptor of telomeric silencing 1‐like (Dot1L) inhibitor blocked transcriptional plasticity, reversing stemness. These findings support that CSCs harbor upregulated chromatin packing domains, contributing to transcriptional and cell plasticity that epigenome modifiers can target.

## Graphical abstract

Through a combination of transcriptomic and histone mapping studies integrated with chromatin imaging, we define how chromatin organization in nanoscale packing domains fine tunes the plasticity of cancer stem cells (CSCs) driving evasion from and survival after chemotherapy. Our results link the physical conformation of chromatin to the transcriptional program of CSCs enabling stemness features and evasion from chemotherapy.

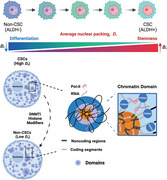

## 1 Introduction

Cancer stem cells (CSCs) represent a rare cell population able to self‐renew, differentiate, and initiate tumors.^[^ ^1^ ^]^ Importantly, CSCs are resistant to chemotherapy and have been implicated in tumor relapse after conventional treatment.^[^ ^2^, ^3^ ^]^ The transcriptional program of CSCs is tightly regulated by epigenetic events and chromatin features which maintain stemness pathways to activate and repress differentiation programs. We hypothesized that the resilience of CSCs under the pressure exerted by cytotoxic treatment is partially driven by their transcriptional malleability, which is facilitated by increased chromatin packing into mass fractal domains. To understand the complex relationship among chromatin conformation, epigenetic states, and transcriptional activity, we integrated nanoscale chromatin imaging with mapping of histone marks to describe drivers of transcriptional plasticity in CSCs versus non‐CSCs.^[^ ^4^ ^]^

Chromatin organizes hierarchically into various structures depending on the length scale:^[^ ^1^ ^]^ at the level of a nucleosome,^[^ ^2^ ^]^ at the level of chromatin domains,^[^ ^3^ ^]^ at the level of topologically associated domains,^[^ ^4^ ^]^ at the level of A/B compartments, and finally^[^ ^5^ ^]^ in chromosome territories. Each structure is thought to regulate transcription and therefore cellular function/phenotype. Previous studies have implicated chromatin packing domains in the regulation of transcription, the capacity of cells for transcriptional reprogramming and differentiation.^[^ ^4^, ^5^, ^6^ ^]^ Recently, nanoscale imaging techniques including optical nanoscopy and electron tomography have revealed that chromatin is organized into several thousand nanoscale packing domains at the level of an individual cell. These packing domains are heritable across cell division, vary in size with an average of 207 kb, and have a mass‐fractal internal conformation where the genomic length *N* scales with the radius of the occupied volume *R* as *N∼R^D^* according to the exponent *D*, which is referred to as the packing density scaling or the mass fractal dimension.^[^ ^4^, ^7^, ^8^ ^]^ Chromatin packing domains have dense heterochromatic cores with chromatin volume concentrations reaching 80% at the center and decreasing toward the periphery.^[^ ^4^, ^6^, ^7^, ^8^, ^9^, ^10^ ^]^ The domain cores tend to be associated with transcriptionally suppressive histone marks H3K9me3 and heterochromatin (H3K27me3), while the periphery is enriched in euchromatic marks H3K4me3 and H3K27ac.^[^ ^9^, ^11^, ^12^ ^]^ The chromatin domain periphery is enriched in RNA polymerases and creates optimal conditions for transcriptional reactions to proceed, including a chromatin volume concentration of ≈35%, which optimizes the diffusion of transcriptional reactants and their binding into transcriptional complexes.^[^ ^7^, ^8^, ^9^, ^13^ ^]^ Accordingly, chromatin domains have been implicated in malignant transformation and chemotherapy resistance.^[^ ^5^ ^]^

It has been hypothesized that alteration of epigenetic marks and DNA methylation patterns delineating active and repressed chromatin play an important role in maintaining CSCs.^[^ ^3^, ^4^, ^14^, ^15^ ^]^ Epigenetic mechanisms of transcriptional reprogramming from a non‐CSC to a “stem‐cell‐like” state, such as TGF‐β‐induced loss of the H3K27me3 mark at the Zeb‐1 promoter has been reported.^[^ ^16^ ^]^ Other factors from the tumor microenvironment (cytokines, hypoxia, metabolic alterations) have also been shown to alter the epigenetic state relative to stemness.^[^ ^17^, ^18^ ^]^ Recent results also suggest that widespread histone modifications in chromatin play a critical role in the global transcriptional reprogramming of cancer cells, including non‐CSCs transitioning to a CSC state.^[^ ^15^ ^]^ Treatment with histone modifiers, including inhibitors of Enhancer of zeste homolog 2 (EZH2) was shown to target CSCs,^[^ ^19^ ^]^ promote cellular differentiation, and inhibit tumor initiation and growth through transcriptional reprogramming.^[^ ^20^ ^]^ These observations support the functional significance of epigenome‐related events in the regulation of transcription in CSCs. Whether agents targeting histone‐modifying enzymes have an impact on chromatin packing and thereby, transcriptional plasticity of CSCs remains undefined.

It is accepted that slow‐cycling CSCs are resistant to chemotherapy and radiotherapy. It has also been shown that under the pressure of chemotherapy, cancer cells acquire stemness features and can convert into CSCs.^[^ ^17^, ^21^ ^]^ Altered chromatin accessibility detectable through ATAC sequencing and mapping of active and repressive histone marks has been associated with chemotherapy‐resistant cellular states,^[^ ^22^ ^]^ which are presumably enriched in CSCs or stem‐like cells. It was reported that upregulation of chromatin packing domains, which may manifest in higher nuclear‐average chromatin packing scaling *D_n_*, was associated with chemotherapy‐resistant states across various cancers and cytotoxic agents,^[^ ^5^, ^13^ ^]^ further supporting the concept that the conformation of chromatin is causally linked to the ability of cells to evade cytotoxic drugs. We posit that the presence of increased numbers of transcriptionally active domains in CSCs is responsible for their ability to withstand chemotherapy.

Here, by using ovarian cancer models, we show that CSCs display upregulation of chromatin packing domains compared to non‐CSCs corresponding to higher transcriptional plasticity in response to cisplatin. Pathways related to Wnt signaling and cytokine–cytokine receptor interaction pathways were repressed through increased deposition of H3K27me3 in non‐CSCs, whereas pathways related to retinol metabolism, antioxidant response, and cytokine signaling were mostly activated and differentially marked by H3K4me3 and H3K27ac in CSCs versus non‐CSCs. Histone modifiers, such as EZH2, DNA methyltransferase, or Dot1L inhibitor (i), downregulated chromatin packing domains and impacted transcription. By downregulating chromatin domains, assessed through the nuclear‐averaged chromatin packing scaling *D_n_*, a Dot1Li attenuated transcriptional plasticity, promoting exit from the stemness state, and restoring response to platinum. In all, our findings support the role of chromatin packing domains regulating the CSC phenotype and associated transcriptional malleability.

## 2 Results

### 2.1 CSCs Exhibit Global Transcriptional Alterations

**Figure 1:**
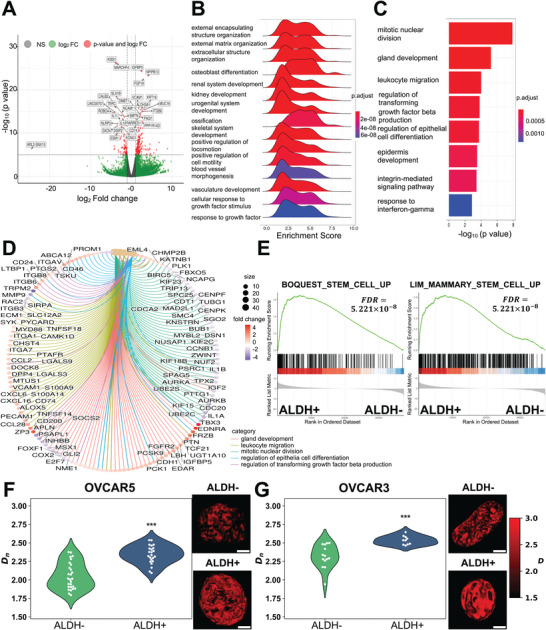
ALDH+ CSCs express stemness pathways and harbor aberrant chromatin organization. A) Volcano plot of differentially expressed genes (DEGs) that compares ALDH+ CSCs derived from OVCAR5 cells with ALDH− non‐CSCs. Genes with a *p*‐value of at most 10^−6^ and a log fold change of at least 2 are shown in red, while genes with expression changes above the same log fold change threshold and *p*‐values larger than 10^−6^ are shown in green. Nondifferentially expressed genes (low log fold change and large *p*‐value) are depicted in gray. B) Gene set enrichment analysis performed using gene ontology (GO) biological process (BP) terms on OVCAR5‐derived ALDH+ CSCs versus ALDH− non‐CSCs. C) −log_10_ (*p*‐value) for the top seven most enriched GO BP terms. Enrichment analysis was performed using the list of DEGs with an adjusted *p*‐value < 0.1. D) Circular network plot of the top five enriched GO BP terms. The sizes of the five nodes corresponding to each BP term are based on the number of DEGs in each term. The colors of each gene node depict the fold change, with red indicating more expression in ALDH+ cells while blue denotes higher expression in ALDH− cells. The colors of the lines connecting the BP term nodes to the gene nodes are based on the term the gene is associated with. E) GSEA for the gene sets BOQUEST_STEM_CELL_UP and LIM_MAMMARY_STEM_CELL_UP shows upregulation in OVCAR5 CSCs versus non‐CSCs (FDR = 0). F,G) Partial wave spectroscopic (PWS) microscopy detects chromatin architecture of CSCs and non‐CSCs derived from OC cells. Representative images display nuclear signals in ALDH+ and ALDH− cells derived from (F) OVCAR5 and (G) OVCAR3. Brighter red indicates higher *D_n_* (scale bar: 5 µm). The violin plots show the distribution of *D_n_* values in the cell population with each individual nucleus shown as a white dot within the violin. Data were collected across three replicates. ALDH+ cells in both cell lines have higher *D_n_* compared to ALDH− cells (*p* < 0.05).

CSCs are characterized by the ability to self‐renew, grow as spheres, differentiate, and generate tumors.^[^ ^23^, ^24^ ^]^ In OC models, cells staining for aldehyde dehydrogenase (ALDH) alone or double staining for ALDH and CD133 have been shown to possess stem cell characteristics.^[^ ^18^, ^25^, ^26^ ^]^ Transcriptomic profiling of ALDH+ versus ALDH− cells identified >500 transcripts significantly differentially expressed (FDR <0.01, fold change >2; **Figure** **1**A). Gene set enrichment (GSE) using gene ontology (GO) terms identified pathways involving extracellular matrix organization, development/differentiation, and response to growth factors as the most enriched in CSCs versus non‐CSCs, whereas mitotic nuclear division was enriched in non‐CSCs (Figure 1B). *Q*‐scores of the enriched pathways are illustrated in (Figure 1C). The molecules driving these pathways and the magnitude of fold change in expression levels are shown in Figure 1D. Gene set enrichment analysis (GSEA) identified stemness‐associated pathways (“Boquest Stem Cells” and “Mammary Stem Cells”) being enriched in ALDH+ versus ALDH− cells (Figure 1E), including genes such as *BMP2*, *BMP4*, *CD24*, *CD44*, and others (Figure S1A, Supporting Information). GO enrichment of upregulated genes revealed that CSCs are specifically upregulating pathways related to development, response to oxidative stress, and processes related to adhesion and migration (Figure S1B, Supporting Information). ChEA analysis identified among the top transcriptional regulators several known transcription factors (TFs) linked to stemness (*SOX2*, CJUN, NRF2, TP63, Figure S1C, Supporting Information). Conversely, CSCs showed downregulation of pathways related to mitotic nuclear division, chromosome segregation, and sister chromatin aggregation, consistent with a less proliferative phenotype of CSCs (Figure S1D, Supporting Information). E2F targets, G2‐M checkpoints, TNF‐a signaling were among the most important downregulated processes (Figure S1E, Supporting Information).

### 2.2 CSCs Harbor Upregulated Chromatin Packing Domains

To determine how the chromatin structure impacts transcription and regulates CSCs and non‐CSC phenotypes, we performed chromatin nanoscale imaging to examine possible differences in the global distribution of chromatin material. Given that exogenous labeling, cellular fixation, and processing can introduce unwarranted modifications to nanoscale chromatin structure,^[^ ^27^ ^]^ we used a live cell chromatin optical spectroscopic nanosensing imaging modality, partial wave spectroscopic (PWS) microscopy, to characterize the native nuclear chromatin organization. PWS microscopy captures label‐free images of nuclear chromatin architecture by collecting the light backscattered by a cell due to variations in density caused by the differential conformation of the chromatin polymer.^[^ ^28^ ^]^ These refractive index variations are directly related to chromatin mass density; therefore, diffraction‐limited images with information of nanoscale chromatin structure between 20 and 200 nm range.^[^ ^29^, ^30^ ^]^ Each PWS microscopy image is presented as a heatmap *D_a_* (*x*, *y*).^[^ ^28^ ^]^ As chromatin is organized in mass‐fractal packing domains, the nuclear average *D_n_* is proportional to the average of the packing scaling and the volume fraction of chromatin domains within the nucleus, which renders *D_n_* a useful metric for assessing chromatin domain upregulation.^[^ ^4^, ^6^ ^]^

PWS microscopy of flow‐sorted OVCAR5 cells demonstrated that ALDH+ CSC populations have higher average nuclear *D_n_* values (*p* = 3.6 × 10^−8^), with the average *D_n_* of the population centered at 2.34, compared to the ALDH− population which was centered around an average *D_n_* of 2.05 (Figure 1F). Higher *D_n_* is a characteristic of malignant cell phenotypes^[^ ^5^, ^13^, ^31^, ^32^, ^33^, ^34^ ^]^ and is strongly correlated with greater transcriptional plasticity.^[^ ^5^ ^]^ Transcriptional plasticity refers to a cell's adaptability of global gene expression patterns in response to its environment and is facilitated by dynamic changes in chromatin structure and domains.^[^ ^9^ ^]^ Increase in *D_n_* corresponds to improved access for transcription factors and RNA polymerase II to DNA material for transcriptional upregulation. We confirmed that this trend is cell line independent by performing PWS microscopy on ALDH+ (mean *D_n_* = 2.53) and ALDH− (mean *D_n_* = 2.32) from OVCAR3 cells (*p* = 3.9 × 10^−4^; Figure 1G). These findings indicate that ALDH+ cells exhibit altered chromatin organization compared to ALDH− cells with chromatin packing domain upregulation, supporting the hypothesis that CSCs harbor an increased ability to reprogram at the transcriptional level, broadening their range of cellular functions or stem‐like behavior.

### 2.3 Histone Modification Mapping Reveals CSC and Non‐CSC‐Profile Differences

**Figure 2:**
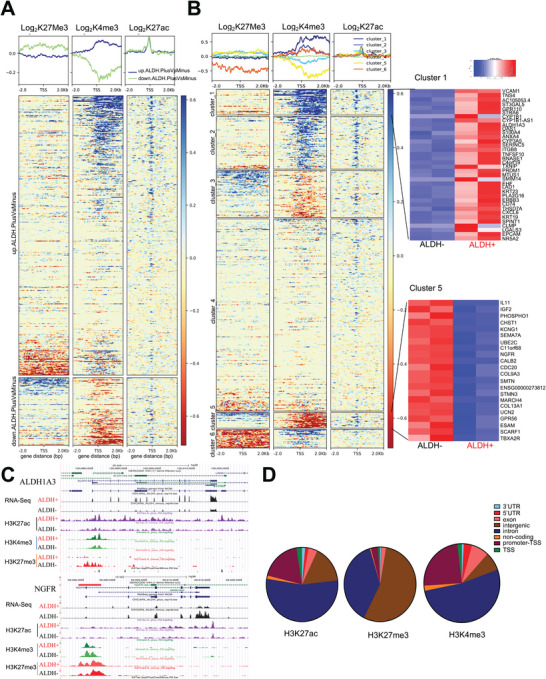
Epigenetic marks mapping in CSCs versus non‐CSCs. A) Heatmaps and metaplots for the log_2_ fold change of the H3K27ac, H3K4me3, and H3K27me3 ChIP‐seq in OVCAR5_ALDH+ CSCs compared to OVCAR5_ALDH− non‐CSCs (*n* = 2). Promoters for all significantly differentially expressed genes are shown, either upregulated in ALDH+ versus ALDH− (blue line) or downregulated in ALDH+ versus ALDH− (green line), as identified in the RNA sequencing (RNA‐seq) analysis. Promoters are hierarchically clustered according to H3K27ac, H3K4me3, and H3K27me3 occupancy. B) The same promoter regions shown in (A) are now *K*‐means clustered (*K* = 6) according to their log_2_ fold change of the H3K27ac, H3K4me3, and H3K27me3 ChIP‐seq in OVCAR5‐derived ALDH+ CSCs compared to ALDH− non‐CSCs (*n* = 2). The average signal for each of these epigenetic marks is shown for all 6 clusters in the metaplots above the heatmaps. Two particularly striking clusters (clusters 1 and 5) are highlighted to the right and accompanied by the RNA‐seq data from the associated genes. C) Representative tracks for H3K27ac, H3K4me3, and H3K27me3 enrichment at the *ALDH1A3* locus in “cluster 1,″, and nerve growth factor receptor (*NGFR*) in “cluster 5.″ Both ChIP‐seq and RNA‐seq tracks are included. D) Genome‐wide distribution of all differentially bound peaks for H3K27ac, H3K4me3, and H3K27me3 in ALDH+ versus ALDH− cells (*p* < 0.05, *n* = 2).

Based on our initial findings of chromatin organization differences between CSCs and non‐CSCs, we conducted CUT&Tag‐sequencing relative to transcriptomic changes in ALDH+ versus ALDH− cells. We observed distinct patterns of H3K27me3 and H3K4me3 deposition for differentially expressed genes (DEGs) in CSCs versus non‐CSCs (adjusted *p* < 0.01). DEGs were then separated into two groups corresponding to “upregulated” (*n* = 397) or “downregulated” (*n* = 96) genes in ALDH+ versus ALDH− cells. We plotted the log_2_ fold change of H3K27me3, H3K4me3, and H3K27ac signals on either side of TSS (±2 kb). Among the upregulated DEGs, we observed a distinct cluster of genes with decreased H3K27me3 in ALDH+ cells. A few of these genes were also enriched in H3K4me3. A large cluster of upregulated transcripts in ALDH+ cells was marked by increased H3K4me3 and most of these genes also harbored increased H3K27ac signal around the TSS (**Figure** **2**A). Among the downregulated DEGs, we identified one cluster of genes with increased H3K27me3 signal (a few of which also had decreased H3K4me3 signal) and a larger, distinct gene cluster which primarily displayed decreased H3K4me3 signals.

To understand how these DEGs were regulated epigenetically, we used *K*‐means clustering and identified subgroups of DEGs with distinct histone modification patterns (Figure 2B). “Cluster 1” included a group of genes marked by increased H3K4me3 deposition in CSCs leading to increased transcription. This cluster also displayed increased H3K27ac deposition at the TSS of genes in CSCs. This group of genes including known stemness‐associated transcripts (*ALDH1A3*, *ERBB3*, *VCAM*, *STRA6*) displayed increased H3K4me3 marks at the TSS in CSCs (Figure S2A, Supporting Information). As representative genes in this cluster, H3K27me3, H3K4me3, and H3K27ac deposition at *ALDH1A3* and *ERBB3* promoters are shown in Figure 2C and Figure S2A (Supporting Information), respectively. Pathway analysis of this cluster identified retinol metabolism, and epithelial development as the most enriched functions and enrichment in pathways governed by *SOX2*, Sox9, Smad‐2, Smad‐3, KLF6 TFs (Figure S2A, Supporting Information) “Cluster 2” was also dominated by increased H3K4me3 and H3K27ac marked genes and included 74 genes upregulated in CSCs such as, *CD74*, *LGALS3*, and *AKR1C3* (Figure S2B, Supporting Information). Interestingly, this group of transcripts is mostly associated with metabolic and antioxidant functions and regulated by *SOX2*, *NANOG*, NRF2, and FOXA1 TFs (Figure S2B, Supporting Information). The distribution of histone marks for representative gene *IDH1* is shown in Figure S2B (Supporting Information). “Cluster 3” included H3K27me3 marked and transcriptionally repressed genes in ALDH+ cells. These genes are related to extracellular matrix and cell migration and immune responses, such as *Col1A1*, *ROBO4*, *SPARC*, and *Aurora kinases A and B* (Figure S3A, Supporting Information). The distribution of histone marks for representative gene *Col1A1* is shown in Figure S3A (Supporting Information). “Cluster 4” includes the largest number of genes (*n* = 269), functionally related to extracellular matrix organization (Figure S3B, Supporting Information). In general, this group of genes does not have detectable differences in histone marks, except for a small subgroup of genes enriched in H3K27me3 and H3K4me3 in CSCs (“poised genes”). The distribution of histone marks for representative gene *MMP2* is shown in Figure S3B (Supporting Information). “Cluster 5” included genes with loss of H3K4me3 marks, downregulated in CSCs, and functionally related to mitosis and cell cycle transition (Figure 2B and Figure S4A (Supporting Information)). Some of these genes also harbored increased H3K27me3 in CSCs. Among them are the cell cycle regulator CDC20, the growth factors nerve growth factor receptor (*NGFR*), IGF2, and GPR receptor GPR56. Decreased transcription of this group of genes in CSCs corresponds to their more dormant phenotype compared to non‐CSCs. The distribution of histone marks for representative gene *NGFR* from this cluster is in Figure 2C. Finally, “Cluster 6” included genes with loss of H3K27me3 in CSCs such as *protocadherin* and *Wnt 10A*, functionally related to Wnt signaling and lipid metabolism pathways (Figure S4B, Supporting Information). The distribution of histone marks for *Wnt 10A* is shown in Figure S4B (Supporting Information). A few genes in this cluster also display increased H3K4me3 around the TSS in CSCs.

### 2.4 CSCs Have Increased Distribution of Poised Chromatin Cores

Having observed differences in chromatin conformation, we further examined the distribution of H2K27me3, H3K4me3, and H3K27ac across the genome. Overall, differential H3K27ac peaks between CSCs and non‐CSCs (*n* = 10141) were mostly distributed in intronic, intergenic, and promoter regions, whereas H3K4me3 peaks (*n* = 995) were found in promoters and introns, and H3K27me3 peaks (*n* = 4438) were detectable primarily in intronic and intergenic regions (Figure 2D).

**Figure 3:**
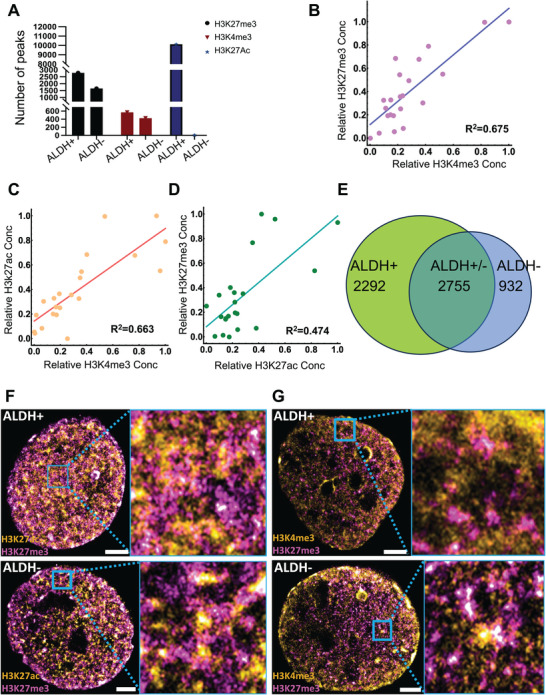
CSCs increase levels of poised heterochromatin cores. A) The total number of differentially bound H3K27ac, H3K4me3, and H3K27me3 peaks in OVCAR5‐derived ALDH+ versus ALDH− cells (*p* < 0.05, *n* = 2). B–D) The correlation between the per‐chromosomal density of (B) H3K27me3 and H3K4me3 (*R* ^2^ = 0.675), (C) H3K27ac and H3K4me3 (*R* ^2^ = 0.663), (D) H3K27me3 and H3K27ac (*R* ^2^ = 0.474). Overall, heterochromatin correlates with euchromatin markers on a per‐chromosome basis. E) To identify poised genes, all called peaks within 2 kb of a TSS were associated with that gene. Genes associated with both H3K4me3 and H3K27me3 are considered “poised” and their numbers in ALDH+ and ALDH− cells are shown. Shared poised genes are included in the overlapping area. F) The spatial distribution of active H3K27ac (yellow) and repressive H3K27me3 (pink), and G) active H3K4me3 (yellow) and H3K27me3 (pink) visualized using stochastic optical reconstruction microscopy (STORM) in ALDH+ and ALDH− cells. Scale bars: 3 µm.

There were more H3K27me3, H3K4me3, and H3K27ac peaks in CSCs versus non‐CSCs (**Figure** **3**A).Interestingly, there was a greater increase in H3K27me3 marks in CSCs than in euchromatin marks (H3K4me3). It has been suggested that in 3D space, heterochromatic cores of chromatin packing domains associate spatially with active transcription to form coupled chromatin clusters.^[^ ^4^, ^8^, ^9^, ^12^ ^]^ Therefore, we investigated if the observed increase in H3K27me3 with elevated transcriptional activity in ovarian CSCs could be due to this pairing process. Utilizing the fact that chromosomal territories represent large, well‐demarcated areas within the nucleus that are associated with cell function and gene regulation, we tested this hypothesis by measuring the per‐chromosomal density of each differentially formed mark in CSCs compared to non‐CSCs (*p*_adj_ ≤ 0.05; sum (bpmark)/chromosome length (bp). In line with the hypothesis that euchromatin and heterochromatin are coupled into domains at larger length scales, we observed a strong correlation between the per‐chromosomal density of H3K27me3 and H3K4me3 (*R* ^2^ = 0.675; Figure 3B) which was comparable to the per‐chromosomal association between both euchromatin marks in both cell types (H3K27ac and H3K4me3, *R* ^2^ = 0.663; Figure 3C and Figure S5A (Supporting Information)). Surprisingly, although H3K27me3 and H3K27ac are antagonistic marks when considered in the context of modification of the same nucleosome or gene segment, we still observed on a per‐chromosomal basis a correlation between H3K27me3 and H3K27ac (*R* ^2^ = 0.474; Figure 3D). Further, we observed that the accumulation of H3K27me3 peaks unique to CSCs was correlated with the increased presence of H3K4me3 peaks (Figure S5A, Supporting Information). As H3K4me3 deposition at the promoter is associated with transcriptional activity, we also analyzed the accumulation of H3K4me3 on the promoter compared to the per‐chromosomal deposition of H3K27me3 and found a strong correlation (Figure S5B, Supporting Information). With the majority of H3K27me3 peaks located in noncoding segments (Figure 2D), this suggested a paired increase in distal packing to facilitate the positioning of promoter sites at domain surfaces for transcription. In the context of bivalent mark formation (H3K4me3/H3K27me3) representing poised states for transcriptional activity, these results suggested that one mechanism of increased transcriptional activity in CSCs could be the result of increased levels of poised chromatin, where the presence of H3K27me3 loci deposited at noncoding (introns, intergenic regions, Figure 2D) interact with distal euchromatin formation to facilitate transcriptional activity. In line with these observations, we examined the numbers of “poised” genes (marked by H3K4me3 and H3K27me3 within 2 kb of all TSS regions in ALDH+ versus ALDH− cells. There were 2.5‐fold higher numbers of poised genes (*n* = 2292) in CSCs versus non‐CSCs (*n* = 932, Figure 3E and Figure S5C,D (Supporting Information)), consistent with this phenotype. The transcription factors regulating pathways enriched among poised genes in ALDH+ cells are functionally related to DNA repair and include BRCA1 (Figure S5F, Supporting Information) and could be involved in the ability of ALDH+ cells to evade chemotherapy. By contrast, TFs involved in regulating poised genes in ALDH− cells are related to stemness, like *NANOG* (Figure S5E, Supporting Information), indicating the potential of these non‐CSC cells to convert to stemness under certain conditions. To visualize the distribution of marked chromatin regions in ALDH+ and ALDH− cells, we employed a multicolor super‐resolution imaging modality, stochastic optical reconstruction microscopy (STORM). The in situ distribution of active (H3K4me3 or H3K27ac) and repressed (H3K27me3) marks is illustrated in (Figure 3F,G). Analysis of H3K27me3 domain cluster sizes is consistent with our previous findings, which show the size of chromatin packing domains to be around 120–200 nm diameter^[^ ^7^ ^]^ (Figure S5G, Supporting Information). As chromatin domains are enriched in RNA polymerase II at their periphery,^[^ ^4^ ^]^ we expected to find active euchromatic marks to be in association with repressive heterochromatin. To this end, we assessed the number of H3K27me3 domain clusters linked to active H3K4me3/H3K27ac‐identified clusters, and vice versa in ALDH+ and ALDH− cells (Figure S5H,I, Supporting Information). A high degree of coassociation among the two groups was observed, with no significant differences between CSCs and non‐CSCs. These findings are consistent with observations from the CUT&Tag analysis and underscore the potential structural interplay at these domain boundaries.

### 2.5 Increased Chromatin Packing Domains in CSCs Correlates with Greater Transcriptional Plasticity

**Figure 4:**
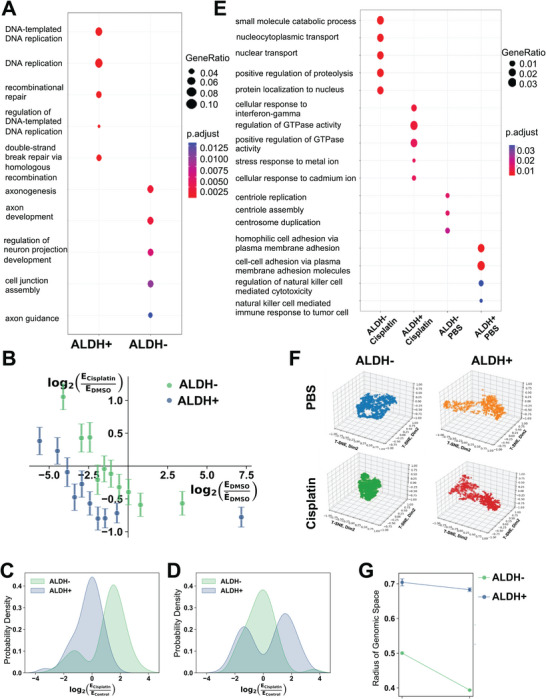
CSCs display greater transcriptional plasticity compared to non‐CSCs. A) GO enrichment analysis indicates the top five biological processes among DEGs (*p* < 0.05) identified through bulk RNA‐seq analysis from flow‐sorted ALDH+ CSCs or ALDH− non‐CSCs treated with cisplatin (1 µm for 24 h) versus control dimethyl sulfoxide (DMSO). B) Change in gene expression as a function of the initial gene expression level. Genes were grouped into quantiles based on the expression level in control (DMSO)‐treated cells. The average expression for control is plotted on the *x*‐axis, while the change in expression after treatment with cisplatin is plotted on the *y*‐axis. Error bars are standard errors of the mean. C,D) Probability distribution functions (PDFs) generated using kernel density estimation (KDE) of the differential expression induced by cisplatin on genes within the DMSO‐treated (control) cells that are underexpressed (C) by ALDH− and those that are underexpressed (D) by ALDH+. E) GO enrichment analysis indicates the top five pathways activated in CSCs versus non‐CSCs at baseline and in response to cisplatin treatment (1 µm, 24 h) based on single cell RNA sequencing (scRNA‐seq) analysis. The differential expression was calculated using pseudobulk values. F) Intercellular transcriptional heterogeneity of control and cisplatin‐treated CSCs versus non‐CSCs. A 3D t‐SNE (stochastic neighbor embedding) dimension reduction was performed on PCA dimensions 1–20 for each condition. Each point represents a cell. G) The radius of genomic space is calculated from the t‐SNE plots in (F) by determining the radius of the circle within which all the cells can be contained.

Given that CSCs exhibit different transcriptomes and baseline differences in chromatin packing domains at baseline, we next sought to examine how this impacts response to a stressor such as cisplatin. Cancer cells have previously been shown to have a higher average nuclear *D_n_* compared to nontransformed cells which allowed more efficient activation of relevant cell survival pathways under chemotherapeutic stress.^[^ ^5^ ^]^ Specifically, subpopulations of cancer cells with high *D_n_* display markers of transcriptional plasticity, including transcriptional malleability, or the ability of a cell to quickly activate stress response genes, and transcriptional heterogeneity, which encapsulates the variety of different pathways that cells can use to evade a stressor.^[^ ^5^ ^]^ As transcriptional plasticity measures the responsiveness of a cell to a stressor, we performed bulk RNA sequencing (RNA‐seq) in CSCs and non‐CSCs treated with cisplatin. Differential gene expression analysis on bulk RNA‐seq data of cisplatin‐treated cells demonstrated that CSCs express DNA replication and repair pathways, while non‐CSCs activate developmental pathways (**Figure** **4**A). High *D_n_* cells tend to show the highest differential expression of genes with low expression due to a higher fraction of chromatin being sequestered into packing domains.^[^ ^13^ ^]^ This allows greater access and binding of transcription factors and RNA polymerase II to promoters at the domain periphery where chromatin volume concentration is optimal for high rates of transcriptional reactions.^[^ ^4^, ^7^, ^36^ ^]^ We tested whether CSCs follow a similar trend by comparing the fold change of gene expression in response to cisplatin relative to the basal expression level of the gene in control‐treated cells. Both CSCs and non‐CSCs displayed the greatest upregulation for underexpressed genes after cisplatin (Figure 4B). Additionally, CSCs tended to show more downregulation of genes after cisplatin, while non‐CSCs mostly upregulated genes under cisplatin pressure (Figure 4B).

Due to the difference in pathways expressed to evade chemotherapy, we analyzed the distribution of the log fold change experienced by genes that are expressed at low levels at baseline in the ALDH− and ALDH+ (Figure 4C,D). We chose to investigate underexpressed genes because the difference in response to chemotherapy treatment was more apparent (Figure 4B). Additionally, computational modeling of the impact of chromatin structure on transcription has shown that changes to *D_n_* have the largest influence on the expression of genes that are initially underexpressed before exposure to a stressor.^[^ ^5^ ^]^ Not only did the CSCs underexpress different genes at baseline, but the direction of differential gene expression upon exposure to chemotherapy was very different compared to non‐CSCs. Genes expressed in the 10th percentile in the ALDH− cells were mostly upregulated after cisplatin treatment, as seen by the probability distribution function (PDF) shifting to the right. By contrast, ALDH+ cells showed little to no change in expression of these genes (PDF centered around zero; Figure 4C). ALDH+ cells, however, showed both upregulation and downregulation of genes that are expressed at low levels at baseline, as indicated by the bimodal distribution of fold change, while ALDH− cells showed no change in expression of these genes after cisplatin exposure (Figure 4D). The magnitude of differential expression due to chemotherapy was similar for both ALDH+ and ALDH− cells, indicating that both cell types may display some transcriptional malleability to the chemotherapeutic stressor. However, the standard deviation of the PDF for differential expression in ALDH+ cells was larger, suggesting that ALDH+ cells may harbor increased transcriptional heterogeneity. In sum, the bulk RNA‐seq data suggested that CSCs harbor more heterogeneous transcriptional expression patterns in response to cisplatin which may enable their survival.

To fully probe whether CSCs are more transcriptionally heterogeneous compared to non‐CSCs, we used single cell RNA sequencing (scRNA‐seq) to examine the spread in the transcriptional program utilized by cells in response to cisplatin. Although there were far more non‐CSCs compared to CSCs in the total cell population, we noticed an increase in the number of reads per cell (35–40k in CSCs vs 3–13k in non‐CSCs) and genes per cell in CSCs (7k in CSCs vs 1–5k in non‐CSCs) (Figure S6A,B, Supporting Information), supporting the hypothesis that CSCs may be more transcriptionally active. To determine whether the increase in gene expression was an artifact of the sequencing protocol, we calculated the number of genes per universal molecular identifier, which showed similarly high values, denoting that most sequenced cells are of good quality to perform further analysis (Figure S6D, Supporting Information). Additionally, there were more cells with high mitochondrial gene expression in the non‐CSCs, suggesting a greater number of apoptotic cells (Figure S6C, Supporting Information). After excluding these cells from analysis, GO enrichment analysis determined which pathways were activated in CSCs versus non‐CSCs. Untreated non‐CSCs showed expression of mitotic genes, while cisplatin‐treated non‐CSCs activated transport pathways (Figure 4E). The untreated CSCs expressed cell adhesion pathways and cytotoxic pathways (Figure 4E), consistent with previous observations from bulk RNA‐seq analysis (Figure 1B). After cisplatin, CSCs showed enrichment of interferon‐gamma, GTPase, and stress response pathways (Figure 4E). To determine whether the entire cell populations in each of the four conditions use the same pathways for survival, we performed an intercellular transcriptional heterogeneity analysis.^[^ ^5^ ^]^ After the t‐SNE dimensionality reduction to three dimensions, we calculated the radius of the sphere within which the cells can be inscribed (Figure 4F,G). Visually, CSCs, both untreated and cisplatin‐treated, were much more spread out whereas non‐CSCs clustered more closely together (Figure 4F). Interestingly, cisplatin treatment did not change the level of intercellular heterogeneity in CSCs, however, non‐CSCs became much less heterogeneous after treatment (Figure 4F), indicating possible refinement of only necessary transcriptional programs to support survival. Quantification of the radius of genomic space confirms these visual observations (Figure 4G). These data suggest that alteration of chromatin structure to prevent transcriptional heterogeneity in CSCs could provide an avenue for improving the efficacy of chemotherapy treatment.

### 2.6 Chromatin Packing Domains are Targetable by Inhibitors of Epigenome Modulators Which Induce Transcriptional Reprogramming in CSCs and Promote Cellular Differentiation

**Figure 5:**
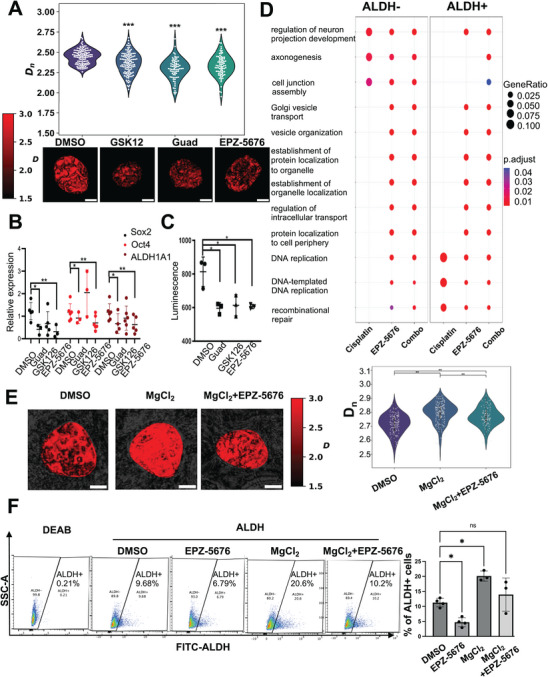
Epigenetic inhibitors reduce chromatin packing domains in CSCs and promote cell differentiation. A) Representative images of chromatin packing domains visualized by PWS microscopy in OVCAR5‐derived ALDH+ CSCs treated with vehicle (DMSO, 0.1%), guadecitabine (100 nm) EZH2i (GSK126, 2 mm), and Dot1Li (EPZ‐5676, 100 nm) for 5 days. The average nuclear *D_n_* is shown below (all scale bars: 5 µm). B) mRNA expression levels of stemness‐associated TFs *SOX2*, *OCT4*, and stemness gene *ALDH1A1* measured by quantitative reverse transcriptase‐polymerase chain reaction (qRT‐PCR) (*n* = 3–4) in OVCAR5‐derived ALDH+ cells treated as in (A). C) Spheroid formation of OVCAR5‐derived ALDH+ CSCs treated with epigenome targeting agents described in (A). Mean values of 3 biological replicates ± standard deviation (SD) are calculated (*p < 0.05; **p < 0.01; ***p < 0.001). D) GO enrichment analysis of the top pathways activated in CSCs and non‐CSCs in response to cisplatin (1 µm, 24 h) alone, Dot1Li (1 µm, 72 h) alone, or priming Dot1Li (1 µm, 72 h) with cisplatin treatment (1 µm, 24 h) compared with DMSO‐treated cells. Transcription was measured by bulk RNA‐seq (*n* = 2 replicates). E) Representative images of chromatin packing domains visualized by PWS microscopy in COV362 cells treated with vehicle (DMSO, 0.1%), MgCl_2_ (5 mm), or MgCl_2_+Dot1Li (EPZ‐5676, 1 µm) for 5 days. The average nuclear *D_n_* is shown (all scale bars: 5 µm). F) Side scatter images and percentages of ALDH+ cells in COV362 cells treated in (DMSO, 0.1%), Dot1Li (EPZ‐5676) (1 µm), MgCl_2_ (5 mm), or MgCl_2_+Dot1Li (EPZ‐5676) were determined by FACS (*n* = 3).

Epigenetic modifications regulate stemness‐related transcriptional programs^[^ ^14^ ^]^ but their impact on chromatin packing domains is not defined. Previous studies have shown that mechanogenomic interventions are able to inhibit new packing domain formation, resulting in chromatin with fewer but more stable domains fostering cell differentiation.^[^ ^6^ ^]^ To test whether epigenetic interventions could have the same result, flow‐sorted CSCs from OVCAR5 and COV362 cell lines were treated with several agents targeting processes involved in packing domain formation, including DNA methyl transferase (DNMT) and histone methyltransferase inhibitors. PWS microscopy measured nuclear *D_n_* in response to treatment with DNA methyltransferase inhibitor (DNMTi) (guadecitabine, 100 nm), EZH2i (GSK126; 2 mm), and Dot1Li (EPZ‐5676, 100 nm) (**Figure** **5**A, schema shown in Figure S7A,B in the Supporting Information). The average nuclear *D_n_* was significantly decreased upon treatment with the epigenome‐targeting drugs (Figure 5A and Figure S7B (Supporting Information)), suggesting that these agents downregulate new chromatin packing domain formation. Indeed, these agents promoted cell differentiation, as demonstrated by a decrease in stemness features, including inhibition of stemness‐associated genes (*SOX2*, *OCT4*, and *ALDH1A1*; Figure 5B and Figure S7C,D (Supporting Information)), and cell renewal ability (spheroid formation assay, Figure 5C) in CSCs derived from OVCAR5 cells and other OC cells (COV362, Figure S7C, Supporting Information; OVCAR3, Figure S7D, Supporting Information). Similarly, DNMT1, EZH2i, and DOT1Li inhibited stemness features in flow‐sorted CSCs derived from human HGSOC tumors (Figure S7E–G, Supporting Information, *n* = 2). Additionally, PWS microscopy demonstrated decrease in *D_n_* in OC cells in which EZH2 or Dot1L were knocked down via shRNA transduction (Figure S7H,I, Supporting Information), supporting that these epigenome targeting enzymes play a role in stemness^[^ ^37^, ^38^ ^]^ and chromatin domain regulation.

As the effects of DNMTi and EZH2i on CSCs had been explored,^[^ ^14^, ^20^, ^35^, ^39^ ^]^ we focused on Dot1Li. Low, noncytotoxic doses of Dot1Li (EPZ‐5676, 100 nm, 5 days) decreased H3K79 di‐ and trimethylation (H3K79Me2 and H3K79Me3) levels in flow‐sorted ALDH+ cells compared with control (Figure S8A, Supporting Information). We performed a similar transcriptional plasticity analysis on Dot1Li‐treated cells sequenced with bulk RNA‐seq as on CSCs versus non‐CSCs. OVCAR5 ALDH+ or ALDH− cells were treated with dimethyl sulfoxide (DMSO), 24 h of cisplatin, 72 h of Dot1Li, or 72 h of Dot1Li followed by cisplatin (denoted as combo treatment) before RNA extraction. After isolating the underexpressed genes in the DMSO‐treated cells and the cells treated with only Dot1Li, the differential expression induced by cisplatin was examined via a PDF plot. The shift in the PDF for Dot1Li‐treated cells suggests that the *D_n_* lowering Dot1Li treatment induced transcriptional reprogramming of both non‐CSCs and CSCs toward differentiation by reducing transcriptional heterogeneity and malleability in response to cisplatin treatment (Figure S8B,C, Supporting Information). Cisplatin induced significant changes in the CSC transcriptome with enrichment in signaling pathways related to DNA replication, DNA template–DNA replication, and recombinational repair compared to non‐CSCs where genes related to axonogenesis, neuron projection development, and cell junction assembly were upregulated (Figure 5D). However, treatment with Dot1Li prior to cisplatin blunted these differences and reduced the enrichment in genes related to DNA repair, rendering the transcriptional program of CSCs to resemble that of non‐CSCs (Figure 5D). Additionally, the Dot1Li blocked stemness associated genes in ALDH+ cells (Figure S8D, Supporting Information), as observed in our previous study.^[^ ^37^ ^]^ These data support the hypothesis that Dot1Li can reduce transcriptional malleability and thereby reprogram CSCs to a nonstem state through reduction of chromatin packing domains.

To demonstrate that the effects of Dot1Li on the stemness phenotype are related to the reduction in *D_n_*, we used a MgCl_2_, in a rescue experiment. Divalent ions have been shown to neutralize negative charges on the DNA promoting assembly of chromatin domains.^[^ ^40^, ^41^ ^]^ Cells incubated with MgCl_2_ for 5 days harbored increased *D_n_*, as measured by PWS, compared to control cells (Figure 5E). Additionally, the Dot1Li‐induced decrease in *D_n_* was rescued by incubation with MgCl_2_ (Figure 5E). At the same time, the ALDH+ cell population was increased in MgCl_2_‐treated cells, as measured by flow cytometry (Figure 5F). Likewise, the Dot1Li‐induced decrease in ALDH+ cells was rescued by incubation with MgCl_2_ (Figure 5F). Together these experiments support the relationship between *D_n_* and stemness.

### 2.7 Dot1L Inhibition Blocks the Formation of the CSC Population In Vitro and In Vivo

**Figure 6:**
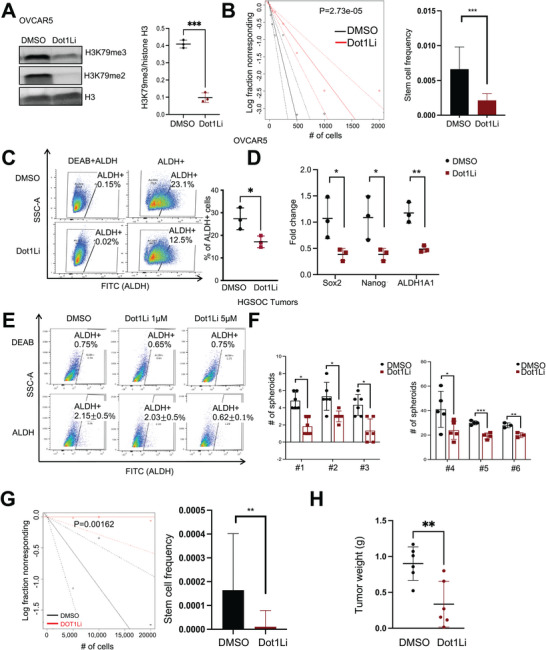
Inhibition of Dot1L reduces OCSC population and stemness gene expression. A) (Left) Western blot analysis of H3K79Me3, H3K79Me2, and H3 (loading control) protein levels in OVCAR5 cells treated with DOT1Li EPZ‐5676 (100 nm, or 1 µm) or DMSO for 5 days. (Right) Quantification is shown (*n* = 3 replicates). B) OVCAR5 cells in a serial dilution (10, 25, 50, 100, 250, 500, 1000, 2000 cells per well) were treated with DMSO or 100 nm Dot1Li for 7 days (*n* = 12). CSC frequency was calculated by using the ELDA software. C) Percentages of ALDH+ cells determined by FACS in OVCAR5 cells treated with 1 mm DOT1Li (*n* = 3). D) mRNA expression levels of *SOX2*, *NANOG*, and *ALDH1A1* in OVCAR5 cells treated with DMSO or DOT1Li (1 µm) for 5 days. Data are shown as means ± SD of 3 biological replicates. E) FACS analysis of the percentages of ALDH+ cells among cell populations derived from human HGSOC tumors and treated with DMSO or DOT1Li (1, 5 µm, 5 days, *n* = 3 biological replicates). F) Quantification of spheroids derived from 1000 cells from human tumor‐derived single‐cell suspensions treated with DMSO or DOT1Li (1 µm) for 5 days, as measured by the CellTiter Glo 3D assay (*n* = 6 tumor specimens). G) Serially diluted DMSO‐ or DOT1Li (1 µm, 5 days)‐treated OVCAR5 cells (500, 1000, and 2500) were injected subcutaneous (SQ) into nude mice to measure tumor initiation and tumor growth. Mice were euthanized and tumors were harvested on day 30 after cell injection. (Left) Log‐fraction plot shows tumor initiation in mice injected with DMSO‐ or DOT1Li (1 µm)‐treated OVCAR5 cells (*n* = 3 per group). (Right) CSC frequencies in the tumors derived from DMSO‐ or DOT1Li (1 µm)‐treated OVCAR5 cells were determined by the ELDA software (http://bioinf.wehi.edu.au/software/elda/) and shown in a bar graph. H) Means ± SD of tumor weights from mice injected with OVCAR5 OC cells treated with DMSO or DOT1Li (1 µm) (*n* = 6 mice per group). Data are shown as means ± SD of replicates, *n* = 5–6 (*p < 0.05; **p < 0.01; ***p < 0.005; and ****p < 0.001).

To further explore the effects of Dot1Li on CSCs, we treated OVCAR5 cells with Dot1Li (EPZ‐5676, 1 µm, 5 days). Dot1Li significantly suppressed the H3K79Me3 and H3K79Me2 levels in OVCAR5 cells (**Figure** **6**A) and reduced cell renewal ability measured as sphere formation under nondifferentiation conditions in a limited dilution assay (Figure 6B; COV362, Figure S8E, Supporting Information and OVCAR3 Figure S8F, Supporting Information) as well as in the ALDH+ population (Figure 6C). Interestingly, the inhibitor did not block the proliferation of the whole cancer cell population (Figure S8G, Supporting Information) or induced apoptosis (Figure S8H, Supporting Information) at the doses tested (100 nm–1 µm). Further, the inhibitor suppressed expression of the stemness associated TFs *SOX2* and *NANOG*, and stemness related genes, *ALDH1A1* in OVCAR5 (Figure 6D; COV362, Figure S8I, Supporting Information; OVCAR3, Figure S8J, Supporting Information). Dot1Li (5 µm, 5 days) significantly inhibited the ALDH+ population from primary HGSOC‐derived cells (Figure 6E and *n* = 3 tumors), and decreased the number of spheroids (Figure 6F), even though there was variability in the capacity to form spheroids across different tumor‐derived primary cells at baseline (*n* = 6 tumor specimens). To further investigate the effects of Dot1Li on tumor initiation capacity in vivo, a limited serial dilution assay was performed in OVCAR5 cells pretreated with DMSO or Dot1Li (EPZ‐5676, 1 µm, 5 days, schema shown in Figure S9A (Supporting Information)). ELDA calculation indicated that Dot1Li‐treated OVCAR5 cells exhibited a lower number of CSCs compared with the DMSO‐treated cells (Dot1Li 1: 94648 vs DMSO 1:6091, *p* = 0.00162; Figure 6G). Dot1Li treatment significantly inhibited tumor initiation (Figure S9B, Supporting Information) and tumor growth at the completion of the experiment (Figure 6H) compared to the control treatment. Flow cytometry analysis of cells dissociated from harvested xenografts indicated that the ALDH+ cell population derived from Dot1Li‐treated OVCAR5 cells was reduced compared with the control tumors (Figure S9C, Supporting Information). Ex vivo treatment with Dot1Li also inhibited the spheroid formation capacity of cells derived from these xenograft tumors (Figure S9D, Supporting Information) compared with control tumors. Consistent with these findings, the transcription of the stemness‐associated genes *OCT4*, *NANOG*, and *ALDH1A1* (Figure S9E, Supporting Information) were suppressed in xenografts derived from Dot1Li‐treated OVCAR5 cells compared with controls. Finally, IHC staining for the stemness‐associated TF *SOX2* demonstrated reduced expression levels in the xenografts formed by Dot1Li‐treated OVCAR5 cells compared to controls (Figure S9F, Supporting Information), suggesting that Dot1Li targets CSCs and prevents tumor initiation.

To further investigate the direct effects of Dot1Li on CSCs in vivo, a subcutaneous (SQ) xenograft model derived from ALDH+ cells was employed. Ex vivo treatment of flow‐sorted ALDH+ cells with Dot1Li (EPZ‐5676, 1 µm, 5 days, Figure S10A, Supporting Information) significantly delayed SQ tumor initiation (Figure S10B, Supporting Information) and tumor growth (Figure S10C, Supporting Information). Importantly, Dot1Li effectively reduced the number of ALDH+ cells (Figure S10D, Supporting Information) and the spheroid‐formation capacity of cells derived from these xenografts compared with DMSO treatment (Figure S10E, Supporting Information). Taken together, these results support the concept that epigenome targeting by Dot1L inhibition alters chromatin organization, resulting in reprogramming of CSCs to nonstem states and inhibiting the key functions of CSCs.

## 3 Discussion

Our results indicate that CSCs harbor upregulated chromatin packing domains and distinct epigenetically marked chromatin regions contributing to a more heterogeneous transcription program that allows stem cells to survive chemotherapy‐related stress. Drugs that inhibit epigenome modifying enzymes, such as Dot1L, downregulate chromatin packing domains and alter the average nuclear *D_n_*, inducing transcriptional reprogramming and exit from stemness and chemoresistant state. Our findings have several implications.

First, for an imaging method free from fixation, cell processing, and labeling, we deployed PWS microscopy as a novel modality of optical spectroscopic chromatin nanosensing.^[^ ^27^, ^42^ ^]^ Here, we show that this method is valuable for studying the dynamic response of CSC populations to drug treatments as well as for ensuring the native chromatin organization remains undisturbed. As CSCs exhibit a nonadherent phenotype, the ability to rapidly image many fields of view allowed for capturing enough cells to analyze population‐level changes. This is vitally important as we observed that CSCs are a somewhat heterogeneous cell population. Given this inherent variability, the use of other methods, such as fixed‐cell, low throughput techniques like electron microscopy would limit the number of cells that can be imaged, increasing the risk of biased results. Altogether, the PWS microscopy imaging deployed here allowed for accurate characterization of chromatin structure within CSC populations.

Second, bulk RNA‐seq results indicated that CSCs and non‐CSCs harbor unique transcriptional signatures. At baseline, CSCs upregulate pathways related to stemness, organ development, and cell migration, while non‐CSCs are enriched in biological processes related to cell cycle and mitotic division. This is consistent with a more proliferative phenotype contrasting the quiescent profile of CSCs. Interestingly, there was an increased number of “poised” gene promoters in CSCs versus non‐CSCs, predicting a more plastic phenotype of stem cells, which would enable them to self‐renew, differentiate, and withstand the toxic effects of chemotherapy. Indeed, increased transcriptional heterogeneity in response to cisplatin was identified by both bulk and scRNA‐seq of ALDH+ cells treated with platinum. Activation of DNA replication and repair pathways was detected in CSCs in response to cisplatin, while non‐CSCs activated gene networks related to axonal development and neuronal differentiation. Activated DNA repair mechanisms have also been reported in CSCs derived from other types of solid tumors in response to chemotherapy, including breast,^[^ ^43^ ^]^ lung,^[^ ^44^ ^]^ and prostate cancer,^[^ ^45^ ^]^ related to enriched stemness features.

Third, we identified distinct organization of histone markings associated with differential transcriptional programs in CSCs. Interestingly, increased H3K27me3 marks were detected in CSCs, suggesting repressed transcription. However, transcription was activated and more malleable in CSCs in response to stressors (cisplatin), contrasting this observation. Increased H3K4me3 and H3H27ac euchromatin marks were also more frequently detected in CSCs and a strong correlation (*R* ^2^ = 0.675) between the per‐chromosomal density of H3k27me3 and H3k4me3 marks was established. This paradoxical finding is consistent with the observed upregulation of chromatin packing domains in CSCs, which contain dense heterochromatic cores and a euchromatic periphery. As most transcriptional processes happen on domain boundaries,^[^ ^4^ ^]^ chromatin domains would be expected to play a key role in the regulation of transcriptionally active and poised genes. Upregulation of chromatin domains suggests the formation of heterochromatic “cores,” consistent with the observed upregulation of these marks. In turn, the transcriptionally active euchromatic periphery of the new domains may help upregulate transcription, which is further supported by the association between euchromatic and heterochromatic marks observed in CSCs (Figure 3). In this context, even as heterochromatin modifications of nucleosomes on a gene body or transcriptional start site would suppress transcription, the observed distal formation of heterochromatin loci may facilitate transcription by forming stabilizing structures around which euchromatin marks are deposited and transcriptional reactions then occur. When considered from the perspective of a highly crowded nucleus, the organization of noncoding elements into high density regions appears to support the ability of coding segments to be efficiently transcribed. As a result, in addition to heterochromatin deposition on TSS of developmental genes restricting differentiation pathways, it can support the malleability of ovarian CSCs by creating many more potential behavior states. Specifically, viewed through the need to both optimize nuclear volume and stabilize diffusion‐limited transcriptional reactions with multiple intermediate complexes, organization into domains creates many new “poised” states. Under conditions of duress, it appears that CSCs are better positioned to sample these poised geometric units more quickly than non‐CSCs. Further, many of the genes stored within the CSCs appear to facilitate stress responses and resistance to chemoresistance. By contrast, non‐CSCs appear to have allocated these poised patterns to optimize the transition into a dedifferentiated CSC state. Such positioning could explain the ability of cancer cells to replenish CSC cells and their capacity for resilience to chemotherapeutic responses.

Finally, we show that epigenome targeting agents such as EZH2, Dot1L, and DNMT inhibitors downregulate chromatin packing domains in CSCs. Importantly, the malleable transcriptional program in response to chemotherapy corresponding to a basal high nuclear *D_n_* in CSCs was prevented by pretreatment with the Dot1Li, one of the epigenome‐modifying agents studied. We validated that DOT1Li inhibited CSCs’ self‐renewal, tumor initiation capacity, and reduced transcriptional heterogeneity in response to chemotherapy. These results support that chromatin targeting is feasible and efficient in CSCs and chemoresistant cells as it leads to cell differentiation, inhibition of tumor initiation, and resensitization to chemotherapy. Other studies have shown effective targeting of CSCs by inhibitors of histone modifying or DNA methylation regulatory enzymes.^[^ ^14^ ^]^ For example, we have shown that treatment with guadecitabine, a potent DNMT inhibitor, suppressed the CSC population and inhibited tumor initiation by inducing cellular differentiation.^[^ ^14^ ^]^ Histone modifiers, such as bromodomain and extraterminal (BET),^[^ ^19^ ^]^ and histone methyltransferase EZH2 inhibitors have also been shown to inhibit CSCs by targeting key pathways regulating stemness.^[^ ^20^ ^]^ Those findings are consistent with our current observations that the EZH2 and the DNMT inhibitor tested here induced a decrease in the average nuclear *D_n_* in ovarian CSCs.

Altogether, our findings link the physical conformation of chromatin detected by PWS microscopy to the transcriptional program of CSCs that facilitates the stemness phenotype and ability to evade chemotherapy. Our findings support further development of PWS microscopy as a potential tool for predicting cancer cell response to CSC‐targeting treatments by assessing the change in chromatin packing domains. Proof of concept for this hypothesis is provided here through the testing and validation of the inhibitory impact of Dot1Li on the nuclear *D_n_* and transcriptional program of ovarian CSCs.

## 4 Experimental Section

### Chemicals and Reagents

Cisplatin (Cat#C2538), and DMSO (Cat#D2650) were from Sigma‐Aldrich. The DOT1Li, EPZ‐5676 (Cat#HY‐15593), was obtained from SelleckChem. The EZH2 inhibitor, GSK126 (Cat#6790) was purchased from Biotechne Tocris (Minneapolis, MN, USA) and the DNMTi, guadecitabine (SGI‐110), was provided by Astex Pharmaceuticals. MgCl_2_ was purchased from Invitrogen (#AM9530G).

### Human Specimens

Deidentified ovarian tumors (*n* = 6) from consenting donors were collected under a Northwestern University IRB‐approved protocol (STU#00202468). Fresh tumors were mechanically and enzymatically disassociated into single‐cell suspensions and cultured as previously described.^[^ ^21^, ^35^ ^]^ Tumor information is listed in Table S1 (Supporting Information).

### Cell Culture

OVCAR5 cells were from Dr. Marcus Peter, Northwestern University. COV362 cells were from Dr. Kenneth Nephew, Indiana University. Cells were maintained in a 37 °C incubator with 5% CO_2_. OVCAR3 cells were purchased from ATCC. Low passage cells were used, and all cell lines were tested to be pathogen and Mycoplasma‐negative (Charles River Research Animal Diagnostic Services). Ovarian cancer cell lines, OVCAR3, OVCAR5, and COV362, were grown under the conditions described in Table S2 (Supporting Information). Stably transduced OC cells with nontargeting shRNA,^[^ ^37^, ^46^ ^]^ shRNA targeting Dot1L^[^ ^37^ ^]^ or EZH2^[^ ^46^ ^]^ were previously described. For imaging, cells were plated on 35 mm glass‐bottom dishes (Cellvis, #D35‐14‐1‐N) treated with poly‐d‐lysine (Gibco, #A3890401) or collagen type I (Millipore Sigma, #C8919).

### Aldefluor Assay and Flow Cytometry

ALDH activity was measured by flow cytometry using an Aldefluor assay kit (Stemcell Technologies, Cat#01700, Cambridge, MA, USA) following the manufacturer's instructions and as described previously.^[^ ^14^, ^21^ ^]^ Cells were sorted using the Fluorescence Activated Cell Sorting, BD FACSAria 5‐Laser Sorter (BD Biosciences, San Jose, CA) and analyzed using the BD FACSDiva Software (v9.0.1, BD Biosciences, San Jose, CA).

### PWS Microscopy for Live‐Cell Imaging

PWS microscopy was a modality of optical spectroscopic nanosensing with diffraction‐limited spatial localization (≈200 nm) and sensitivity to the statistical properties of chromatin conformation from the scale of the 20 nm chromatin chain to the size of chromatin packing domains (200–300 nm).^[^ ^4^, ^28^ ^]^ Spectroscopic nanosensing took advantage of the difference between spatial resolution and the detectability of subdiffractional length scales. For a given location within a nucleus, the refractive index was proportional to the local chromatin density, which was in turn linked to the 3D conformation of the chromatin polymer. The spectrum of interference between a reference wave and waves scattered from all spatial refractive index variations within the coherence volume defined by the spatial coherence in the transverse plane and the depth of field longitudinally was determined by the autocorrelation function of chromatin density with the range of sensitivity from 20 to 300 nm. The standard deviation of the interference spectra was proportional to the Fourier transform of the autocorrelation function integrated over the Fourier transform of the coherence volume. PWS microscopy imaging was performed using a microscope design that was previously published.^[^ ^13^, ^28^ ^]^

The PWS microscopy system was built into an inverted commercial microscope (Leica DMIRB) and images were captured through a 63× oil immersion objective lens with a Hamamatsu Image‐EM CCD camera C9100‐13 coupled with a liquid crystal tunable filter (LCTF; Cri Woburn, MA). Cells were illuminated using light from an Xcite‐120 LED Lamp (Excelitas, Waltham, MA) that was passed through the LCTF programmed to take images at 2 nm intervals for wavelengths between 515 and 685 nm. All cells were imaged in physiological conditions (37 °C and 5% CO_2_) using a stage‐top incubator (TOKAI HIT, INU Incubation System for Microscopes). PWS acquisition captured a 3D image cube I(*λ*, *x*, *y*), where each (*x*, *y*) pixel contained the backscattered light intensity filtered by wavelength (*λ*) captured across the 10 000 µm^2^ field of view.^[^ ^28^ ^]^ An image cube was also captured in a region of the culture dish without any cells to use as a reference scattering spectrum.

### PWS Microscopy Image Analysis

High‐frequency noise from LED illumination was reduced by applying a low‐pass Butterworth filter on the PWS microscopy image cube. Analysis of the image cubes began with calculating the standard deviation in (*x*, *y*) pixel intensity across wavelengths, which was then used to calculate the local average chromatin packing scaling *D_a_* (*x*, *y*) given a priori known optical parameters of illumination and signal acquisition and the functional form of the chromatin density autocorrelation function previously measured by chromatin electron tomography.^[^ ^4^, ^7^, ^10^ ^]^ For a given cell, the nuclear average *D_n_* could be calculated by averaging *D_a_* (*x*, *y*) across the nucleus. Local average *D_a_* (*x*, *y*) and nuclear average *D_n_* were proportional to the average of the packing scaling and the volume fraction of chromatin domains within a given coherence volume and the whole nucleus, respectively. In other words, a higher *D_n_* was a measure of the upregulation of chromatin domains. A lower *D_n_* typically corresponded to the nuclear phenotype with fewer but larger and more mature domains.^[^ ^4^, ^6^ ^]^ Violin plots for *D_n_* values were generated using the seaborn Python package.^[^ ^47^ ^]^ Representative PWS images were generated by converting pixel values in the raw images to *D_a_*, then applying a custom red colormap to nuclear regions of interest. The nonnuclear regions were shown in grayscale.

### In Vivo Xenograft Experiments and Extreme Limiting Dilution Analysis

For tumor initiation experiments, serially diluted DMSO‐ or DOT1L‐inhibitor (1 µm)‐treated OVCAR5 cells (5000, 10 000, and 20 000) were mixed with an equal volume of Matrigel (Corning) and subcutaneously injected into female athymic nude mice (6–8 weeks old, Envigo). Length, width, and depth of tumor xenografts were measured with a digital caliper twice a week. Tumor volume was calculated with the formula volume = ½ × length × width × depth. The ELDA software (http://bioinf.wehi.edu.au/software/elda/) was used to estimate CSC frequencies in harvested tumors.^[^ ^28^ ^]^ Another SQ xenograft model used 10 000 DMSO‐ or DOT1L‐inhibitor (1 µm)‐treated OVCAR5‐derived ALDH+ CSC cells (*n* = 3) for examining the direct effects of DOT1L inhibitor on the tumorigenicity and tumor growth of CSCs (*n* = 3). The animal experiments in this study were approved by the Northwestern University Institutional Animal Care and Use Committee (IACUC, protocol #IS000003060).

RNA extraction, quantitative reverse transcriptase‐polymerase chain reaction (RT‐PCR) analysis, Western blotting, immunohistochemistry, RNA sequencing and analysis, spheroid formation, cell proliferation and apoptosis assays, and multicolor super‐resolution STORM imaging, were performed as previously described^[^ ^21^, ^48^, ^49^ ^]^ and are included in the Supporting Information.

### Intracellular Transcriptional Plasticity Analysis

Raw gene counts were converted to transcripts per million (TPM) using RSEM,^[^ ^50^ ^]^ as TPM normalization accounted for both sequencing depth and the impact of gene length on the number of mRNA reads. TPM data from RSEM were imported using the tximport R package^[^ ^51^ ^]^ and converted to scaled count values for further analysis of transcriptional plasticity using Python. First, the dataset was filtered to remove External RNA Controls Consortium gene identifiers and any genes with NaN values. Next, total TPM values for each sequenced sample were normalized to have a sum of 1 by dividing individual gene values by the sum of all genes within the respective samples. The resulting transcript fraction values were multiplied by 1 × 10^6^ to convert values back to TPM. The dataset was then filtered to remove genes that had a value of 0 in any of the sequenced samples before combining the two replicates using averaging. The log_2_ fold change between genes in the control group and the cisplatin‐treated group was calculated and used to filter out cells with an absolute fold change of less than 1. The expression values of these genes in the control group were organized into 10 percentiles and the genes in the lowest percentile were extracted. For comparing CSCs to non‐CSCs, the log_2_ fold change was calculated for these genes between control and cisplatin‐treated ALDH+ cells and between the same groups in the ALDH− cells. In the comparison of Dot1Li‐reprogrammed CSCs to normal CSCs, the log_2_ fold change was calculated between control and cisplatin‐treated ALDH+ cells for normal CSCs, while the fold change between Dot1Li‐ and combination (Dot1Li+cisplatin)‐treated was used for reprogrammed CSCs. The resulting values were used to create a kernel density estimate (KDE) plot using the seaborn Python package^[^ ^47^ ^]^ with default parameters.

### CUT&Tag

The CUT&Tag sequencing was performed with the CUT&Tag‐IT Assay Kit (Active motif, 53160) by following the manufacturer's protocol. Briefly, 10^5^ OVCAR5 ALDH+ CSCs and OVCAR5 ALDH− non‐CSCs were FACS‐sorted and incubated with Concanavalin A beads for binding. Cells were then incubated with a primary antibody targeting H3K27me3 (Active Motif, 39155), H3K4me3 (Abcam, ab8580), and H3K27ac (Active Motif, #39034) overnight at 4 °C. Samples were then incubated with secondary antibody, followed by CUT&Tag‐IT‐assembled pA‐Tn5 Transposomes. After tagmentation at 37 °C for 1 h, DNA was purified and cleaned up by SPRI beads. DNA was then amplified with a combination of i7 and i5 Indexed primers for library preparation. The quality of libraries was checked by High Sensitivity DNA Assay (Agilent Technologies) and sequenced on NextSeq 2000 sequencer. Raw sequencing data were converted into fastq files which were run through the NF‐Core/cutandrun pipeline v3.1 (https://doi.org/10.5281/zenodo.5653535). In short, raw paired‐end 50 bp sequencing reads were checked with FastQC (v0.11.9), trimmed with TrimGalore (v0.6.6), and mapped to human genome build GRCh38 using Bowtie2 (v.2.4.4) with standard settings. Peaks were called using MACS2 (v2.2.7.1) callpeak with cut‐off FDR (*q*‐value) at 0.05. Differentially bound peaks were also identified with Homer getDifferentialPeaksReplicates.pl in histone mode. UCSC Genome Browser was used to visualize locus‐specific signals. To study the histone marks near the differentially expressed genes, canonical transcript coordinates from the UCSC genome browser were downloaded for DEGs previously described in the RNA‐seq methods. The promoter coordinates for these genes (defined as 2 kb up‐ and downstream of the canonical TSS) were calculated, and deeptools (v3.5.1) was used for visualization and clustering of the changes in histone marks in these promoters. Raw read data and genome browser tracks could be found in the National Center for Biotechnology Information (NCBI) Gene Expression Omnibus (GSE268169).

Per‐chromosome analysis of differentially increased CUT&Tag peaks was performed. The total segment length of each mark (H3K27ac, H3K27me3, H3K4me3) that was uniquely increased in CSC compared to non‐CSCs was calculated. Marks were included if their *p*‐value was less than 0.1 in this analysis. The summed segment length was then normalized to the chromosome total length and pairwise comparisons were performed. For statistical analysis, a pairwise linear regression was calculated as reported within the main text figures. Owing to the distinct mechanisms of heterochromatin formation in chromosome X, this was excluded from this form of analysis. CSC and non‐CSC differential peaks as well as total peaks were analyzed to understand the differences between cell types as well as the functional coupling between broad increases in domains in the context of euchromatin and heterochromatin deposition. Finally, analysis of H3K4me3 deposition at the transcription start sites compared to H3K27me3 accumulation was performed due to the role of H3K4me3 in transcriptional activation at the gene promoter.

### scRNA‐seq and Analysis

ALDH± cells were FACS‐sorted from OVCAR5 cells treated with PBS or cisplatin (1 µm, 24 h), and further processed for single‐cell sequencing libraries preparation. Sequencing libraries were created using a Chromium Next GEM Single Cell 3ʹ Kit v3.1 (10X Genomics, Cat. PN‐1000269) targeting 10 000 cells per sample according to manufacturer's instructions and pooled in equimolar quantities. Premade scRNA‐seq libraries were sequenced by NovaSeq S2 PE50 Sequencer. FASTQ files were aligned to GRCh38 using 10X Genomics Cellranger version 7.1.0 on a computing cluster. The aligned files with read counts were read into R using the Seurat v5 package^[^ ^52^ ^]^ for analysis. A Seurat object was created for each of the conditions with the minimum number of cells set to three and the minimum number of features set to 200. The percentage of mitochondrial DNA was calculated for each cell by filtering for genes labeled with “MT” and comparing it with the total number of genes expressed by the cell. The Seurat objects were filtered to remove cells with less than 20 000 and more than 100 000 mRNA reads, which indicated membrane compromised or dead cells with low expression and cell doublets, respectively. Additionally, cells with 15% or more mitochondrial DNA expression were removed, as these cells were likely to be undergoing cell death. Differential gene expression analysis was performed on the remaining cells after preprocessing by pseudobulking to create gene expression profiles for each sample that were then input into DESeq2. Data could be found in the NCBI gene expression omnibus (GEO) (GSE268169, GSM8285239‐GSM8285242).

### Intercellular Transcriptional Heterogeneity Analysis

The gene expression from filtered Seurat objects was log normalized with a scale factor of 10 000 before using a VST method to find variable features, which were then used to scale each Seurat object. PCA analysis was done on the scaled Seurat objects using the variable features. Dimensions 1–20 from the PCA data were used for a 3D t‐SNE analysis. The final t‐SNE results were weighted based on the cell count for each condition and exported from R for further analysis in Python. As a measure of intercellular transcriptional heterogeneity, the radius of genomic space for each treatment group was calculated in Python as previously described^[^ ^5^ ^]^ using the formula $R_{c}=\sqrt{\frac{1}{N}\sum_{i=1}^{N}{\left(r_{i}-r_{\mathrm{mean}}\right)}^{2}}$ where *N* is the total number of cells, *r_i_* is the position of each cell, and *r*_mean_ is the average position of all cells.

### Statistical Analysis

All experiments were done in at least biological duplicates. Data were analyzed by Student's *t‐*test, one‐way ANOVAs with Tukey's multiple comparisons test, or two‐way ANOVAs with Sidak's multiple comparisons test. *p*‐values from ANOVAs were multiplicity‐adjusted *p*‐values. All statistical analyses were done using GraphPad Prism software. For all the panels, a *p*‐value less than 0.05 was considered significant (*p < 0.05, **p < 0.01, ***p < 0.001). Outliers were selected by using the Outlier Calculator (GraphPad) and a significance level of 0.05. For PWS microscopy experiments, statistical analysis was performed by using the open‐source Python mathematics package SciPy to run a Student's *t*‐test^[^ ^53^ ^]^ to evaluate significance among different conditions using a significance threshold of 0.05/*N*, where N is the number of groups. Analysis of significance was taken between the unpaired and unequal variance of average nuclear *D_n_*, normalized by the *D_n_* of the untreated control group against the different conditions.

### Ethical approval

Use of deidentified human specimens from consenting donors were collected under a Northwestern University IRB‐approved protocol (STU#00202468). The animal experiments in this study were approved by the Northwestern University IACUC (protocol #IS000003060).

## Conflict of Interest

The authors declare no conflict of interest.

## Author Contributions

Y.W. and J.F. contributed equally to this work. Y.W., J.F., V.B., and D.M. designed the experiments; Y.W., J.F., K.I.M., L.M.A., Y.Z., G.W., H.H., C.L.D., I.T., and J.L. performed experiments; J.F., E.T.B., I.C.Y., A.D., P.C.G., J.P., J.C., Z.J., and M.A. completed bioinformatic analyses; Y.W., J.F., K.I.M., L.M.A., G.W., and R.G. analyzed data; Y.W., J.F., E.T.B., V.B., and D.M. wrote and edited the paper; V.B. and D.M. provided funding for the study.

## Supporting information



Supporting Information

## Data Availability

The data that support the findings of this study are openly available in NCBI GEO (GSE268169) at https://www.ncbi.nlm.nih.gov/geo/query/acc.cgi?acc=GSE268169, reference number 268169.
