# Supplementary material for: Chromatin Organization Governs Transcriptional Response and Plasticity of Cancer Stem Cells
Source: Adv Sci (Weinh). 2025 Mar 7;12(17):2407426. doi: 10.1002/advs.202407426 (PMC12061297; doi:10.1002/advs.202407426)
Supplement: Supplementary file 1 — Supporting Information [file ADVS-12-2407426-s001.pdf]

ADVANCED SCIENCE
Open Access

Supporting Information

for *Adv. Sci.*, DOI 10.1002/advs.202407426

Chromatin Organization Governs Transcriptional Response and Plasticity of Cancer Stem Cells

Yinu Wang, Jane Frederick, Karla Isabel Medina, Elizabeth Thomas Bartom, Luay Matthew Almassalha, Yaqi Zhang, Greta Wodarcyk, Hao Huang, I Chae Ye, Ruyi Gong, Cody Levi Dunton, Alex Duval, Paola Carrillo Gonzalez, Joshua Pritchard, John Carinato, Iuliia Topchu, Junzui Li, Zhe Ji, Mazhar Adli, Vadim Backman and Daniela Matei**

Supplemental Material

Supplemental Material and Methods:

Spheroid formation assay: Ovarian cancer cells derived from human tumors were pre-treated with DMSO (control), guadecitabine (100nM), GSK126 (2μM), and Dot1Li (1μM) inhibitors for 5 days. Cells were cultured (1000/well) in MammoCult medium in 96-well low-attached plates (Fisher Scientific, Cat#3474) for 7-14 days as described in our previous studies (1). Viable cells were quantified by measuring intracellular ATP levels with the CellTiter-Glo 3D cell viability assay (Promega, Cat#G9681) following the manufacturer's protocol as we described previously (2). Luminescence was measured by using a microplate reader (SpectraMax GeminiXS, Molecular Devices, San Jose, CA, USA). Serial diluted numbers of OC cells (0, 25, 50, 100, 250, 500, 1000, 2000/well) were seeded into low-attached 96 well-plate and cultured in MammoCult medium (with DMSO or 100nM Dot1L inhibitor) for 7 days. There were 12 replicates per condition. CSC frequency based on spheroid formation was calculated by using the ELDA software (http://bioinf.wehi.edu.au/software/elda/).

Cell proliferation and apoptosis assays: Cells (OVCAR5, OVCAR3 and COV362) were seeded on 96-well plates at 2000-3000 cells/well, treated with DMSO or Dot1Li (100nM, 500nM and 1μM) and cultured in an IncuCyte S3 live-cell analysis system. For cell proliferation, four images of bright field/well were captured at 12 hours intervals for 6 days. For apoptosis, Annexin V Green Dye (Sartorius, cat. no. 4642) was used for detection of apoptotic cells. Four images of bright field and green fluorescence were captured at 3 hours intervals for 72 hours after treatment. Cells confluency and FITC green florescence intensity were recorded and used for assessing apoptosis.

RNA extraction, quantitative RT-PCR analysis: Total RNA was isolated with Trizol reagent (Invitrogen, Carlsbad, CA) or RNeasy Micro kit (Qiagen, #74004) according to the manufacturer's instructions. RNA quantity and purity (260/280 absorbance ratio) were determined using a NanoDrop spectrophotometer (Thermo Scientific). For mRNA expression studies, 1μg of total RNA was reverse transcribed into cDNA with an iScript cDNA synthesis kit (Bio-Rad, Berkeley, California) per manufacturer. cDNA was used for quantitative PCR performed by using the iTaq Universal SYBR Green Supermix (Bio-Rad, Berkeley, California) on a 7900HT real-time PCR instrument (Applied Biosystems, Foster City, CA). The PCR reactions used the following parameters: 94°C for 10 min, 40 cycles of amplification at 94°C for 15 s and 60°C for 1 min, and an extension step of 7 min at 72°C. Data were normalized using the expression of the 18S gene. Relative expression of target genes was calculated using the $2^{-\Delta(\Delta C_T)}$ method where $\Delta C_T = C_{T,\ target} - C_{T,\ 18S}$, and $\Delta(\Delta C_T) = \Delta C_{T,\ stimulated} - \Delta C_{T,\ control}$. Primer sequences (Integrated DNA Technologies, USA) are in Supplemental Table S3.

Western Blotting: Cell lysates were prepared in radioimmunoprecipitation assay (RIPA) buffer. Protein concentrations were quantified with the Bradford assay (Biorad Protein Assay Reagent, BioRad, CA). Proteins (20-50μg/sample) were denatured at 100°C, resolved by polyacrylamide-gel electrophoresis (SDS-PAGE), and transferred onto a PVDF membrane. The membrane was incubated with 5% BSA (blocking), and then with primary antibody overnight at 4°C, followed by incubation with HRP-conjugated secondary antibody for 1hr at room temperature. The signal was developed using SuperSignal West Pico PLUS Chemiluminescent Substrate (Thermo Fisher Scientific cat#: 34580) and captured with an ImageQuant LAS 4000 machine. To detect additional proteins, membranes were treated with Restore Western Blot Stripping Buffer (Thermo Fisher

Scientific cat#: 21059), blocked, and then incubated with primary antibody. Primary and HRP-conjugated secondary antibody information are listed in the Supplemental Table S4.

Immunohistochemistry (IHC): Sections of paraffin-embedded xenograft tissues, were deparaffinized with xylene, followed by re-hydration through decreasing concentrations of ethanol (100%, 90%, 70%, 50%, 0%). Antigen retrieval was performed with citrate buffer (10mM, pH 6.0) for 30 minutes at 95°C as previously described (3). Peroxidase activity was eliminated with 10% hydrogen peroxide (Fisher Scientific, Cat# H324500) for 10 mins, then tissues were incubated with 0.5% normal goat serum (DAKO, Hamburg, Germany, Cat# K0672) in PBS for 1 hour, followed by primary antibodies (anti-Sox2, Invitrogen, Cat# 20G5 MA1-014, 1:100 dilution, or mouse IgG antibody, Santa Cruz, Cat# sc-2025, 10mg/ml) overnight-incubation at 4°C. Then tissues were treated with avidin-biotin peroxidase reagents of a DAKO Detection Kit (DAKO, Hamburg, Germany, Cat# K0672) and with liquid DAB substrate chromogen (DAKO, Cat# K3467). Sections were counterstained with hematoxylin (Agilent Technologies, Cat# CS700) and cover slipped.

Bulk RNA sequencing (RNA-seq): The RNA-seq libraries include FACS sorted OVCAR5 derived ALDH+ CSCs vs. ALDH- non-CSCs at baseline, and ALDH+/- CSCs/non-CSCs treated with DMSO, Dot1Li (500nM, 48 hours), cisplatin (1µM, 24 hours) and Dot1Li+cisplatin (n=2 per experimental group). For each sample, 100ng of total input RNA was extracted by Rneasy Micro Kit (Qiagen, Cat#74004). mRNA was isolated by NEBNext Poly(A) mRNA Magnetic Isolation Module, and RNA-seq libraries were prepared using the NEBNext Ultra II RNA library prep kit from Illumina (New England Biolabs Inc., Ipswich, MA). All the RNA-seq libraries were checked by using a BioAnalyzer (Agilent Technologies) and then sequenced on an Illumina HiSeq4000 system with single-end, 50-bp read length settings.

Bulk RNA-seq data analysis: For quality control, raw fastq files were pre-processed using TrimGalore (v0.6.6) and cutadapt (v2.10) with single-end trimming mode, Phred score cutoff of 30, and minimum sequence length cutoff of 20 bp (4). After checking quality with the FastQC tool (v0.11.9), raw sequencing reads were aligned to human genome build hg38 using STAR (5) v.2.5.2 (https://github.com/alexdobin/STAR) with standard settings. Mapped reads were converted to raw gene counts with HTSeq v.0.6.1 (6). Differentially expressed genes were identified with the R package EdgeR (7) with an adjusted p-value cut-off of 0.01. NGSCheckMate (8) was used to validate cell line identity by genotyping RNA-seq reads and comparing them to reference RNA-seq data from the Cancer Cell line Encyclopedia (9). Data can be found in the NCBI Gene Expression Omnibus (GEO) (GSE268169). Gene set enrichment analysis for gene ontology biological process terms was conducted using the R packages clusterProfiler (10, 11) and Enrichplot. The differentially expressed gene lists were analyzed with GSEA (12, 13), and Ingenuity Pathway Analysis (IPA, QIAGEN) with standard settings, to identify differentially expressed pathways. Transcription Factor Protein-Protein Interaction (TF-PPI) pathway enrichments were analyzed using Enrichr (https://maayanlab.cloud/Enrichr/) (14-16).

Multi-color super resolution STORM imaging and Analysis: For nanoscale imaging of poised genes, OVCAR5 derived ALDH+ and ALDH- cells were plated onto 8-well glass bottom chambers coated with collagen type I and fixed in 4% paraformaldehyde. After fixation, quenching was performed with 0.1% sodium borohydride (#CAT: 452882, Millipore Sigma) in PBS. After washing, cells were blocked in 3% bovine serum albumin (BSA, #CAT: A9647, Sigma) and permeabilized with 0.2% Triton X-100 (#CAT: T8787, Millipore Sigma) for 1 hour at RT. STORM labeling was performed overnight at 4°C using primary antibodies for H3K27me3 (Abcam, ab192985), H3K4me3 (Abcam, ab213224), and H3K27ac (Thermo Fisher, MA5-23516).

Following three 5-minute washing in buffer (0.2% BSA, 0.1% Triton X-100 in PBS) at RT, cells were incubated with secondary antibodies Alexa Fluor 647 (Thermo Fisher, A21245) and Alexa Fluor 488 (Thermo Fisher, A32723) for 1 hour at RT (29). The STORM optical setup was constructed using a commercial inverted microscope platform (Eclipse Ti-E with Perfect Focus System, Nikon). Illumination was provided by two continuous lasers: a red laser with a 637 nm emission (OBIS Laser Box, Coherent) and a second green laser with a 532 nm emission (MGL-FN-532, Changchun New Industries Optoelectronics Tech. Co., Ltd.). The lasers were collimated, delivering an average power of 3 to 10 kW/cm^3 at the sample. Imaging was performed using a 100× objective with a 1.49 numerical aperture (SR APO TIRF, Nikon) and recorded by an electron-multiplying CCD camera (iXon Ultra 888, Andor) to capture a 10,000-frame acquisition. The raw image stack was first processed in ImageJ by subtracting its minimum projection. Subsequently, the rolling ball background subtraction tool was applied using a rolling ball radius of 5 pixels for all images in the stack. The preprocessed stack was then analyzed using the Thunder-STORM plugin in ImageJ. The camera configuration for this plugin was set according to the imaging parameters, which included a pixel size of 130 nm (calculated as camera pixel size divided by objective magnification), photoelectrons per A/D count as 18.6, base level [A/D counts] as 0, and an EM gain of 240. Given the localization-based nature of the STORM data, we employed Density-Based Spatial Clustering of Applications with Noise (DBSCAN) to analyze the H3K27me3 data. The DBSCAN parameters were set as $\epsilon = 50$ nm, $n = 3$. The size of H3K27me3 clusters was determined by calculating the area of the convex hull formed by the clustered points. This area was then normalized by a factor of $\pi \times (80\ nm)^2$ and filtered between 0.1 to 100. To quantify the association between H3K27me3 and H3K4me3/H3K27ac, we calculated the percentage of H3K27me3 clusters, with which H3K4me3/H3K27ac colocalized within 30 nm of the cluster boundary, as the association of H3K27me3 cluster to H3K4me3/H3K27ac. Similarly, the association of H3K4me3/H3K27ac with H3K27me3 clusters was calculated as the percentage of H3K4me3/H3K27ac colocalizing within 30 nm of any H3K27me3 cluster.

Supplementary Table S1:
Patients' characteristics.

	Tumor Type	Treatment History	Site
Pt #1	recurrent ovarian cancer, BRCA negative (stage IIIC)	3 cycles of neoadjuvant chemotherapy treatment: carboplatin/taxol followed by 3 cycles of dose-dense carboplatin/abraxane	ascites (paracentesis)
Pt #2	high-grade serous carcinoma (stage IIIC)	4 cycles of neoadjuvant chemotherapy (carboplatin + taxol)	ovarian tumor
Pt #3	high grade serous carcinoma – (Stage IIIB)	neoadjuvant chemotherapy (carboplatin + taxol)	ovarian tumor
Pt #4	grade 3 clear cell adenoarcinoma (stage IIIC)	chemotherapy-naïve	ovarian tumor
Pt #5	high-grade serous carcinoma (stage IVB)	3 cycles of neo-adjuvant chemotherapy: carboplatin/taxol	peritoneal tumor
Pt #6	clear cell carcinoma (stage IVB)	chemotherapy-naïve	omental tumor

Supplementary Table S2.
Cell culture media

Cell line	Culture medium
OVCAR3	RPMI-1640 (ATCC, Cat# 30-2001) 20% FBS (Corning, Cat# 35011CV)+ 1% penicillin/streptomycin + 0.01mg/mL insulin human recombinant (Gibco catalog number: 12585-014)
OVCAR5	RPMI 1640 (Corning, Cat# 35011CV), 10% fetal bovine serum (FBS) (Corning, Cat# 35011CV), 1% penicillin/streptomycin (Corning, Cat# 30-002-CI), and 1X GlutaMAX (Gibco, Cat# 35050061)
COV362	Dulbecco's Modified Eagle Medium (DMEM) (Corning, Cat#10-017-CV), 10% FBS, 1% penicillin/streptomycin, 1X GlutaMAX.

Supplementary Table S3:
Primer sequences for selected genes in qRT-PCR analysis.

Gene Name	Primer Sequence	
	Forward (5’ to 3’)	Reverse (5’ to 3’)
ALDH1A1	ATCAAAGAAGCTGCCGGGAA	GCATTGTCCAAGTCGGCATC
ALDH1A3	CACAGGTTGCCTTCCAGAGG	TGGCTTCCCTGTATCCATCG
NANOG	ACATGAGTACTGCTTTAGTTGGT	TCCACCCCAACCAAAAATTTAACA
OCT4	GTAGTCCCTTCGCAAGCCC	GGGGCGAGAAGGCGAAAT
SOX2	AGGATAAGTACACGCTGCCC	TAACTGTCCATGCGCTGGTT
18S	CGT CTG CCC TAT CAACTTTC	GATGTGGTAGCC GTTTCTC

Supplemental Table S4. Antibody information

Antibodies	Source	Dilution	Cat#
Anti-Histone H3 (tri methyl K79)	Rabbit polyclonal	1:1000	Abcam Cat# 2621
Anti-Histone H3 (di methyl K79)	Rabbit monoclonal	1:1000	Abcam Cat# 177184
Anti-Dot1L	Rabbit polyclonal	1:1000	Abcam Cat# 64077
Anti-EZH2	Mouse monoclonal	1:1000	Cell Signaling Cat#3147
Anti-H3K27me3	Rabbit polyclonal	1:1000	Cell Signaling Cat# 9756
Anti-Histone H3	Rabbit polyclonal	1:4000	Abcam 1791
HRP-conjugated donkey-anti-rabbit antibody	Donkey polyclonal antibody	1:2000	GE Healthcare Cat# NA9340
HRP-conjugated goat-anti-mouse antibody	Goat polyclonal	1:2000	R&D System Cat# haf007
HRP-conjugated goat-anti-rabbit	Goat polyclonal	1:2000	BioRad Cat# 1706515

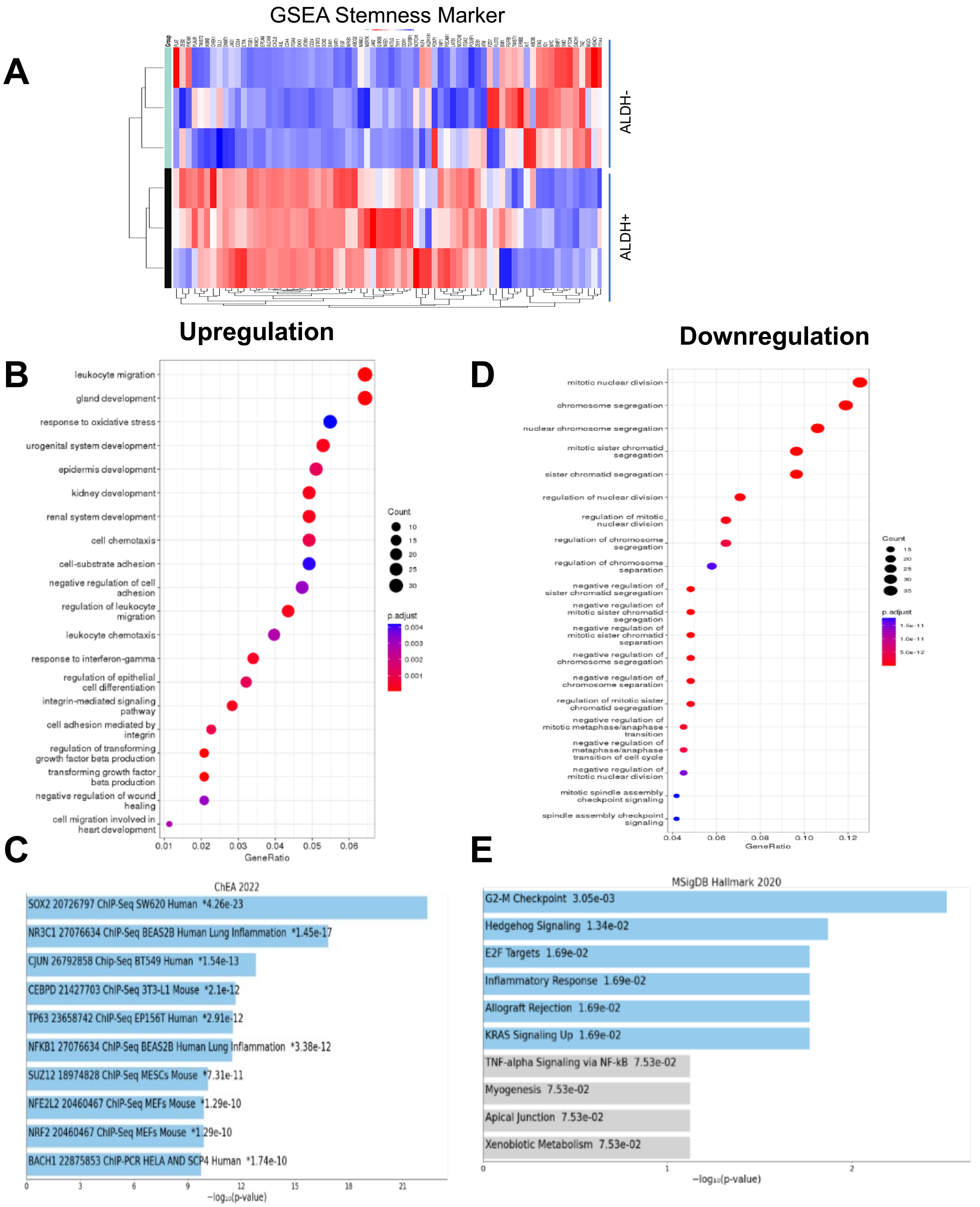

Supplemental Figure S1. (A) Heatmap of differentially expressed genes included in the GSEA MALTA_CURAED_Stemness_Markers in COV362 derived ALDH+ cells vs. ALDH- cells. Transcription levels were measured by RNA seq (n=3 replicates/group). (**B)** Dot plot of over-representation analysis (ORA) of DEGs shows the top 20 pathways enriched among upregulated and **(D)** downregulated DEGs between ALDH+ vs. ALDH- cells FACS sorted from OVCAR5 cell line. The size of the circles represents DEG counts within each term, and the color of the circles represents statistical significance. Gene ratio (x-axis) is the relative number of DEGs per term. **(C)** ChEA analysis of the top transcription factors regulating enriched pathways among upregulated DEGs between ALDH+ vs. ALDH- cells. **(E)** GO analysis for Biological Process identifies pathways enriched among downregulated DEGs in ALDH+ vs. ALDH- cells. Gene expression was measured by RNA-seq (n=2 replicates per group).

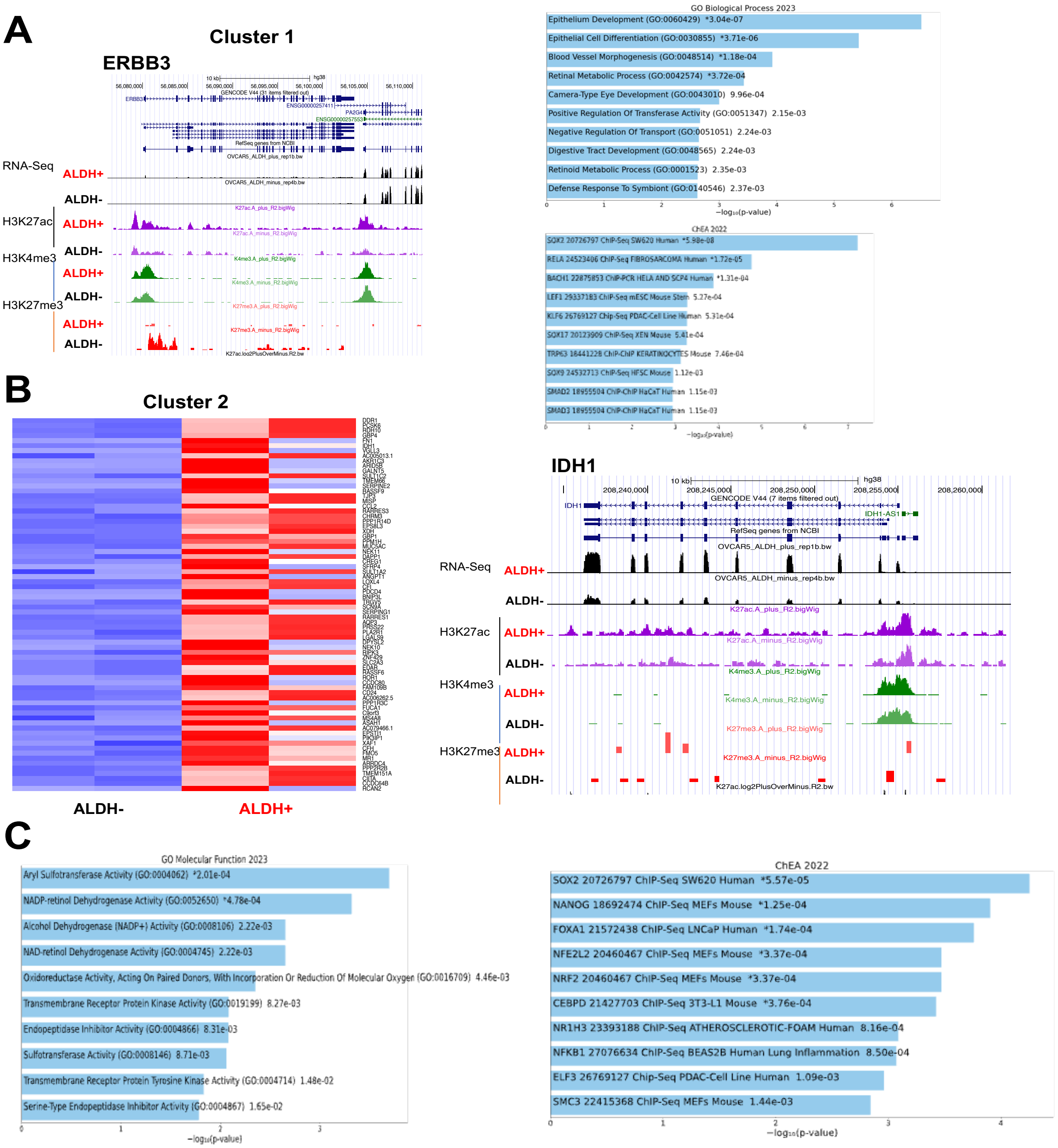

Supplemental Figure S2. (A) Representative RNA seq tracks and Cut&Tag sequencing tracks for histone marks H3K27ac, H3K4me3 and H3K27me3 enrichment at ERBB3 locus in ALDH+ and ALDH- cells (Cluster 1). GO Analysis for Biological Process and ChEA of the top pathways and TFs enriched among the Cluster 1 genes. **(B)** (**Left**) Heatmap of DEGs in ALDH+ vs. ALDH- in Cluster 2. (**Right**) Representative RNA seq tracks, Cut and Tag sequencing tracks for histone marks H3K27ac, H3K4me3 and H3K27me3 enrichment at isocitrate dehydrogenase 1 (IDH1) locus in OVCAR5 derived ALDH+ and ALDH- cells (Cluster 2). **(C)** (**Left**) GO Analysis for Molecular Function and **(Right)** ChEA analysis of DEGs in ALDH+ CSCs vs. ALDH- non-CSCs shows the top enriched pathways and TFs among DEGs in Cluster 2. Gene expression was measured by RNA-seq (n=2 replicates per group).

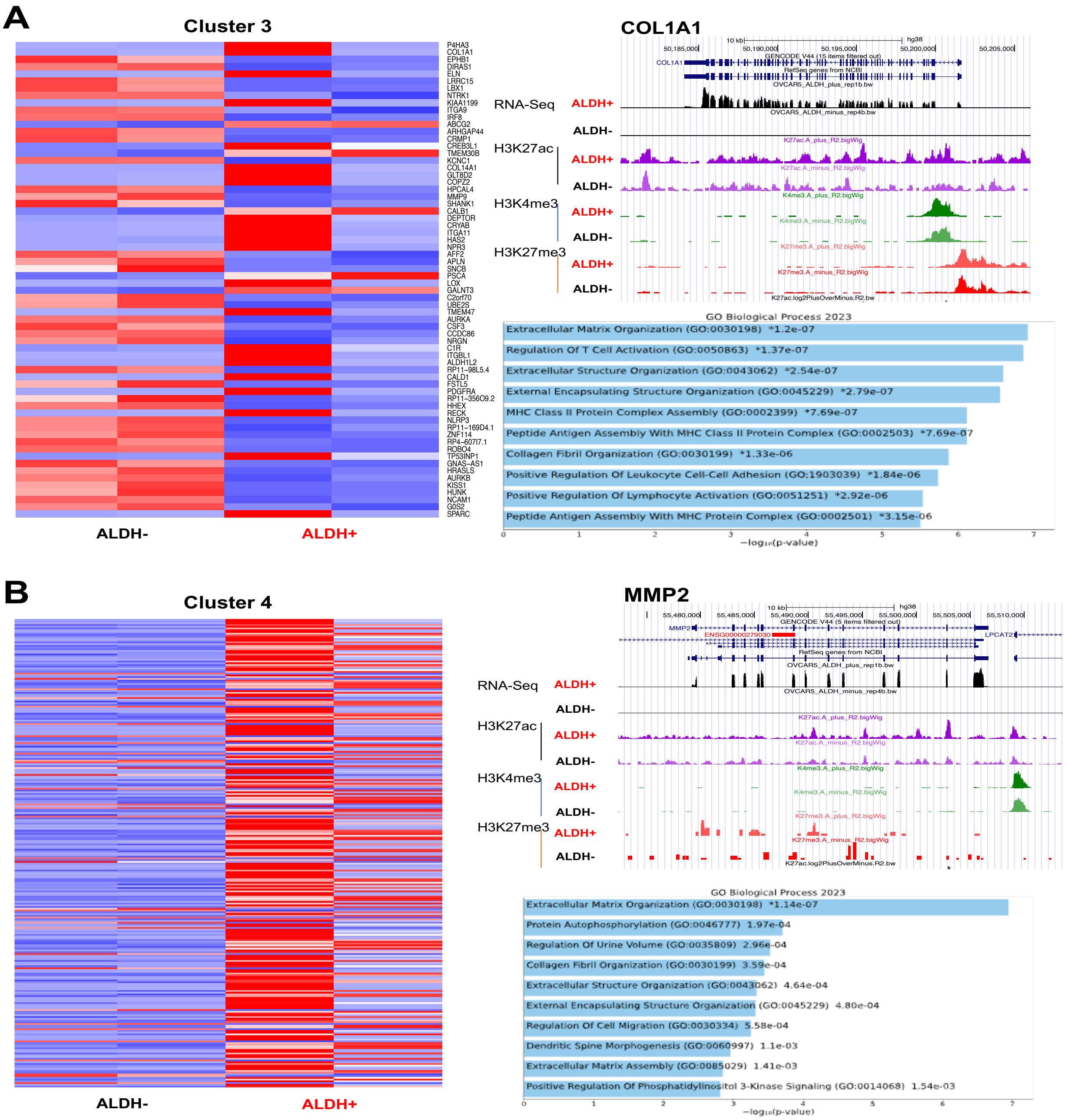

Supplemental Figure S3. (A) **(Left)** Heatmap for DEGs in OVCAR5 derived ALDH+ vs. ALDH- cells in Cluster 3. Gene expression was measured by RNA seq (n=2 replicates/group). **(Right upper)** RNA seq tracks and Cut and Tag sequencing tracks for histone marks H3K27ac, H3K4me3 and H3K27me3 at collagen type 1, alpha 1 (COL1A1) locus in ALDH+ and ALDH- cells (Cluster 3). **(Right lower)** GO Analysis for Biological Processes among DEGs ALDH+ vs. ALDH- cells shows the top enriched pathways in Cluster 3. **(B)** **(Left)** Heatmap for DEGs in OVCAR5 derived ALDH+ vs. ALDH- OC cells in Cluster 4. **(Right upper)** RNA seq tracks and Cut and Tag sequencing tracks for histone marks H3K27ac, H3K4me3 and H3K27me3 at matrix metallopeptidase 2 (MMP2) locus in ALDH+ and ALDH- cells (Cluster 4). **(Right Lower)** GO analysis for Biological Processes among DEGs in ALDH+ vs. ALDH- cell indicates the top enriched pathways in Cluster 4.

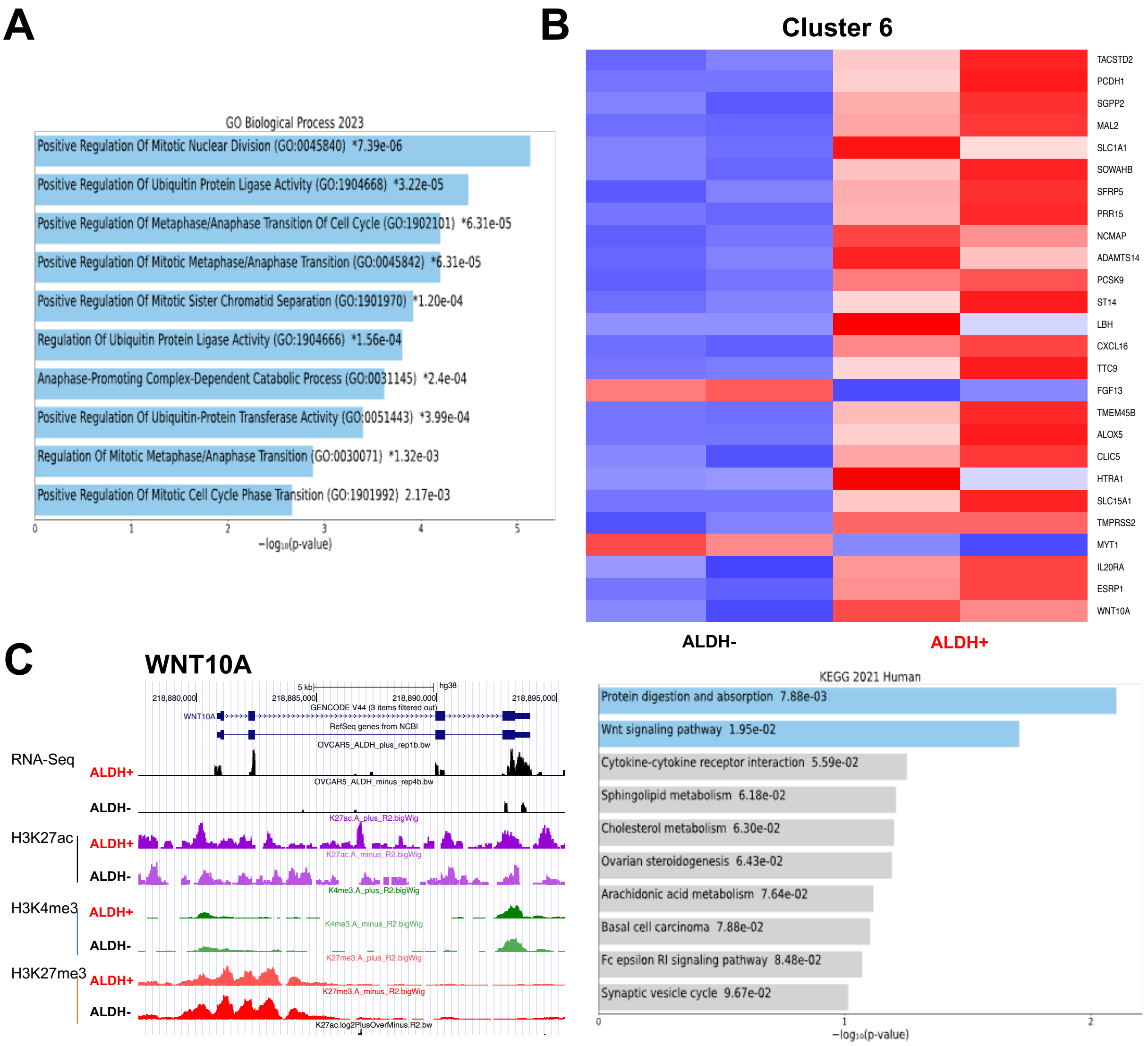

Supplemental Figure S4. (A) GO Analysis (Biological Process 2023) indicates the top pathways enriched in ALDH+ CSCs vs. ALDH- non-CSCs in cluster 5. **(B)** Heatmap for DEGs in OVCAR5 derived ALDH+ vs. ALDH- OC cells in cluster 6. (**C**) **(Left)** Representative tracks for H3K27ac, H3K4me3 and H3K27me3 enrichment at the Wnta10A locus in "Cluster 6" in ALDH+ and ALDH-cells. (**Right)** Pathways activated in ALDH+ CSCs vs. ALDH- non-CSCs in Cluster 6, analyzed by KEGG. Gene expression was measured by RNA seq (n=2 replicates per group).

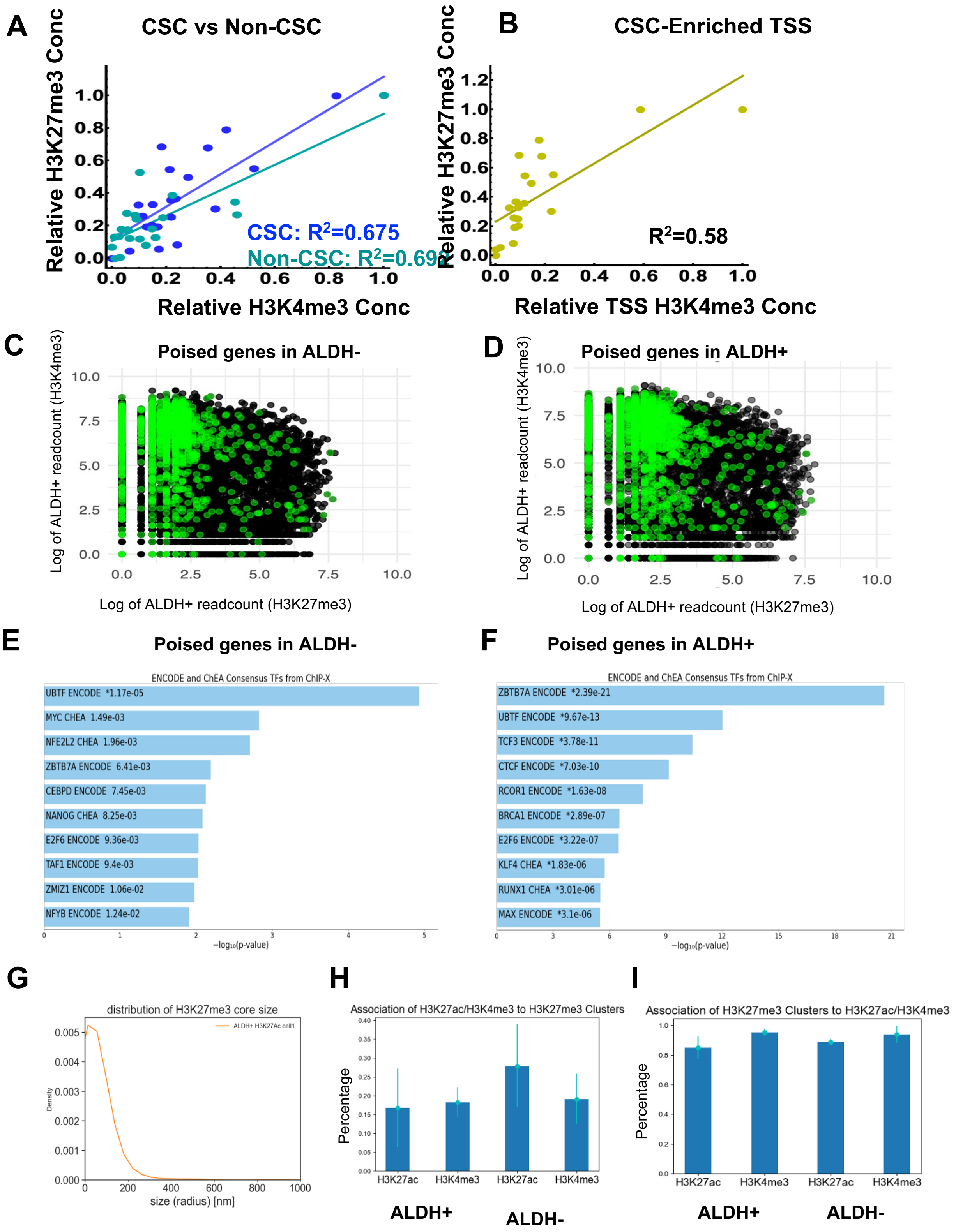

Supplemental Figure S5. (A) Analysis of histone modifications (H3K4me3, euchromatin) and heterochromatin (H3K27me3) peaks unique to CSC and non-CSC cells. In both cell types,

euchromatin levels increase with the total deposition of heterochromatin. As H3K27me3 peaks increase in CSCs, there is a concordant increase in H3K4me3 at the global scale. **(B)** Analysis of H3K4me3 deposition on gene promoters compared to the total deposition of H3K27me3 indicates that the deposition of euchromatin marks on gene promoters is associated with distal compaction. **(C)** To identify global patterns in co-occupancy of these marks, coordinates for canonical TSSs for all human genes were identified and the log of H3K4me3 vs. H3K27me3 read counts were plotted for all promoters (TSS +- 2kb, pseudo count of 1, n=1) for ALDH+ CSCs and **(D)** ALDH-non-CSCs. Green dots indicate "poised" genes identified in ALDH+ cells as described in (**Fig. 3E). (E)** ENCODE and ChEA Consensus TFs from ChIP-X indicate the top TFs involved in regulating the poised genes identified in ALDH- and in **(F)** ALDH+ cells. **(G)** Domains identified by STORM imaging using DBSCAN revealed that the average K27me3 domain size, calculated as the distance from the center to the outer edge, ranged from 120 to 200 nm in size **(H)** Active euchromatin marks (H3K27ac and H3K4me3) co-localized with repressive marks (H3K27me3) **(I)** Repressive heterochromatin H3K27me3 marked domains, exhibited a high degree of association with euchromatin marks (H3K27ac and H3K4me3).

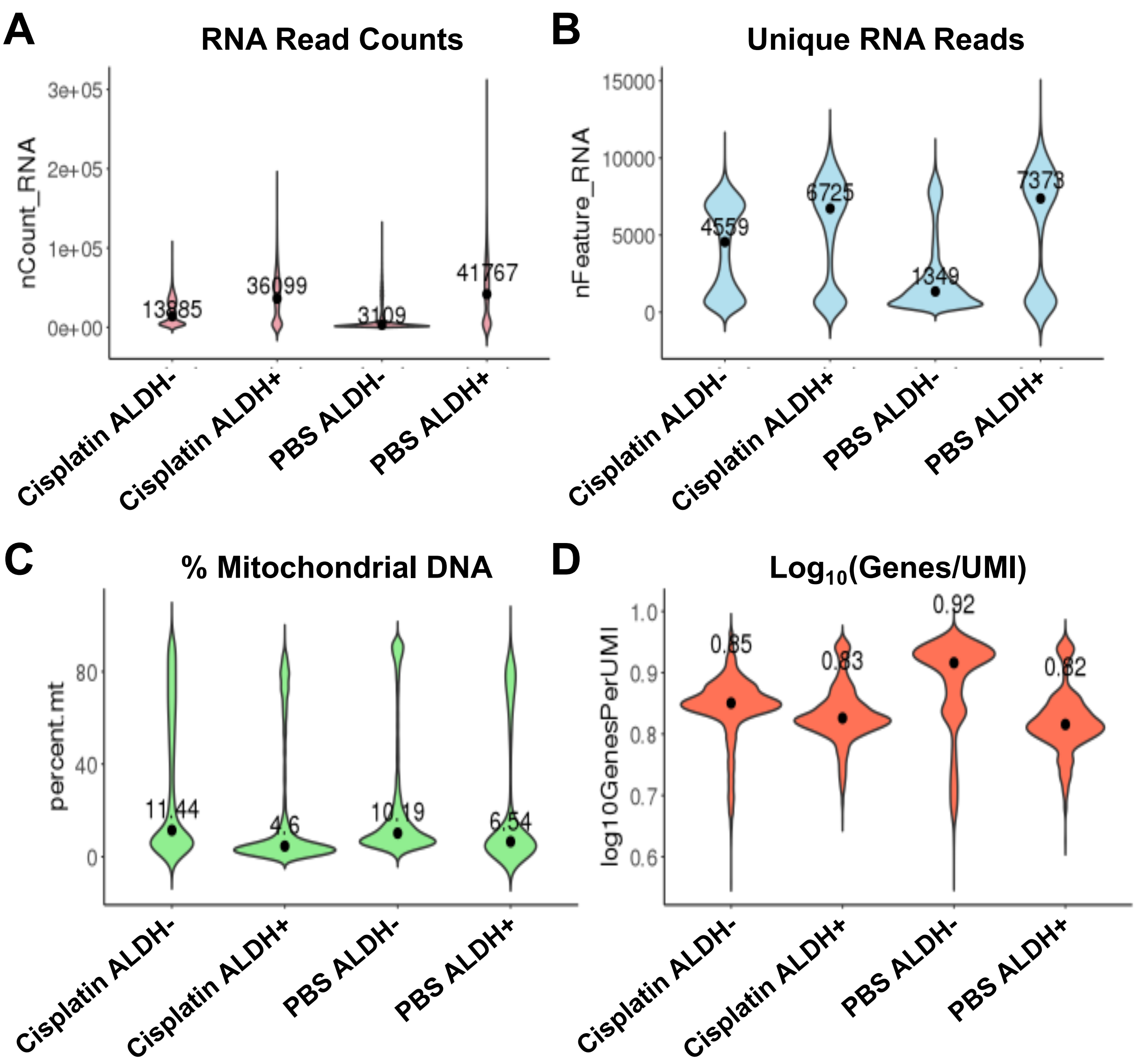

Supplemental Figure S6. Analysis of raw scRNA-seq sample characteristics. **(A)** The total number of RNA reads identified via sequencing for each cell in the listed conditions. **(B)** The number of unique RNA reads mapped to genes found for each cell. **(C)** The percent of mitochondrial DNA expressed within each cell indicates whether a cell has undergone cell death. **(D)** The number of identified genes per unique UMI bar code (representing individual cells) displays the total number of genes sequenced for each cell.

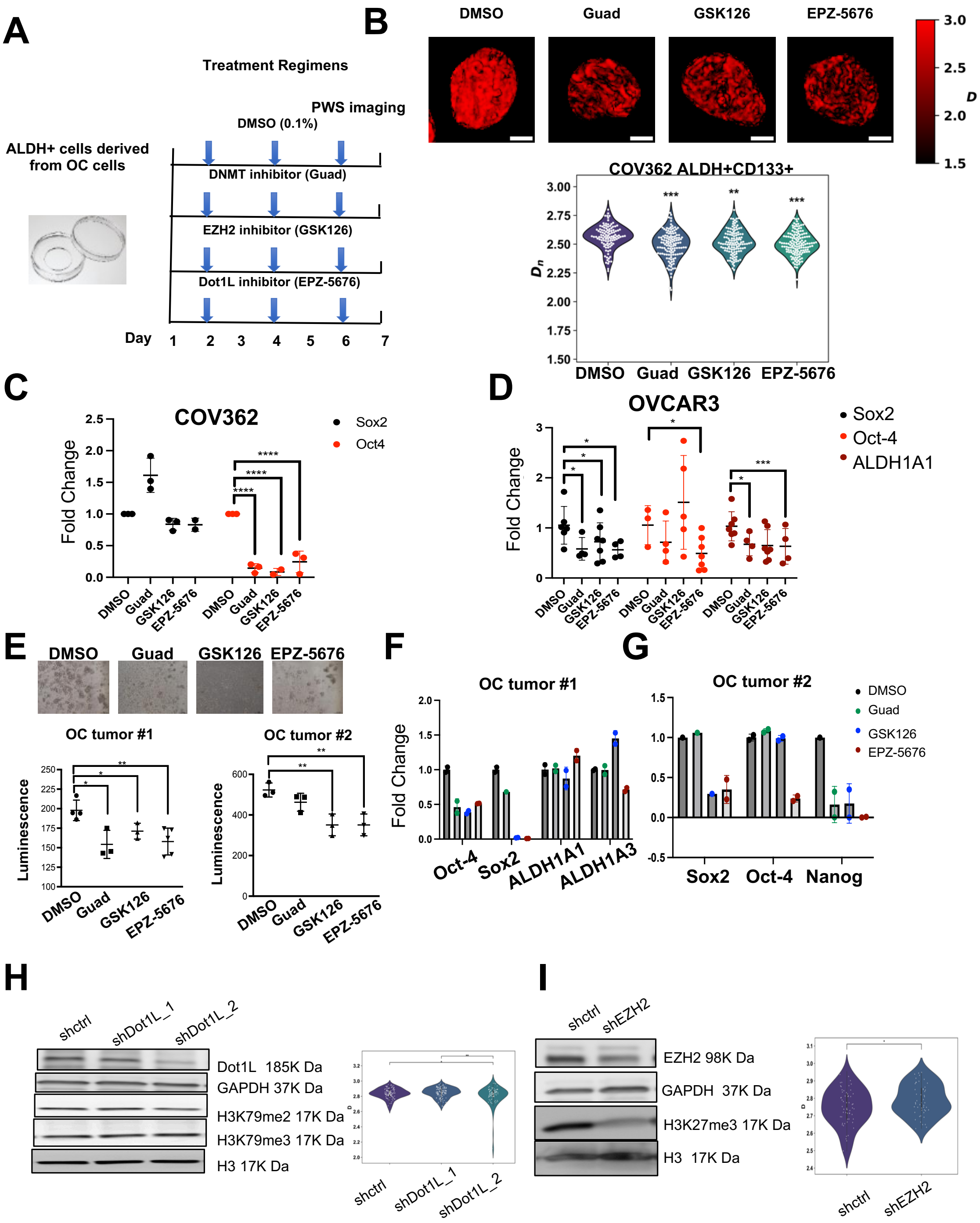

Supplemental Figure S7. Epigenetic inhibitors reduce chromatin packing domains in CSCs and promote cell differentiation. (A) The schema shows the treatment schedule of ALDH+ CSCs prior to imaging. FACS sorted ALDH+ CSCs were seeded on fibronectin or poly-D-Lysine pre-coated plates, treated with vehicle (DMSO, 0.1%), guadecitabine (100nM), GSK126 (2mM) and EPZ5676 (100nM) for 5 days prior to imaging. **(B)** Representative images of chromatin packing domains visualized by PWS in COV362 derived ALDH+ CSCs treated with vehicle (DMSO, 0.1%), guadecitabine (100nM), EZH2i (GSK126, 2mM), and Dot1Li (EPZ5676, 100nM) for 5

days. The average nuclear D_n is shown below (All scale bars: 5μm). **(C)** mRNA expression levels of stemness associated TFs *SOX2, OCT4,* and stemness gene ALDH1A1 measured by qRT-PCR (n=3-4) in COV362 derived ALDH+, and **(D)** OVCAR3 derived ALDH+ CSCs treated as in **(A). (E)** Spheroid formation of HGSOC primary tumor-derived ALDH+ cells treated with epigenome targeting agents described in (A) (n=2). Quantification of spheroids was measured with the CellTiter Glo 3D assay. **(F-G)** mRNA expression levels of stemness associated TFs *SOX2, OCT4, NANOG* and stemness gene *ALDH1A1, ALDH1A3* measured by qRT-PCR (n=3) in ALDH+ CSCs derived from HGSOC tumor treated as in **(A)** (n=2). **(H)** (Left) Western blot measured Dot1L, H3K79Me3, H3K79Me2, and loading controls (GAPDH and histone H3) protein levels in COV362 cells stable transduced with shRNAs targeting Dot1L (shDot1L) or control shRNAs (shctrl). shDot1L_1 and shDot1L_2 represent different shRNA sequences. (Right) Representative images of chromatin packing domains visualized by PWS in these cells are shown. The average nuclear D_n is shown below (All scale bars: 5μm**). (I)** (Left) Western blot measured EZH2, H3K27Me3, and loading controls (GAPDH and histone H3) protein levels in SKOV3 cells stable transduced with shRNAs targeting EZH2 (shEZH2) or control shRNAs (shctrl). (Right) Representative images of chromatin packing domains visualized by PWS in these cells are shown. The average nuclear D_n is shown below (All scale bars: 5μm) **(***, P <0.05; **, P< 0.01; ***, P< 0.005).

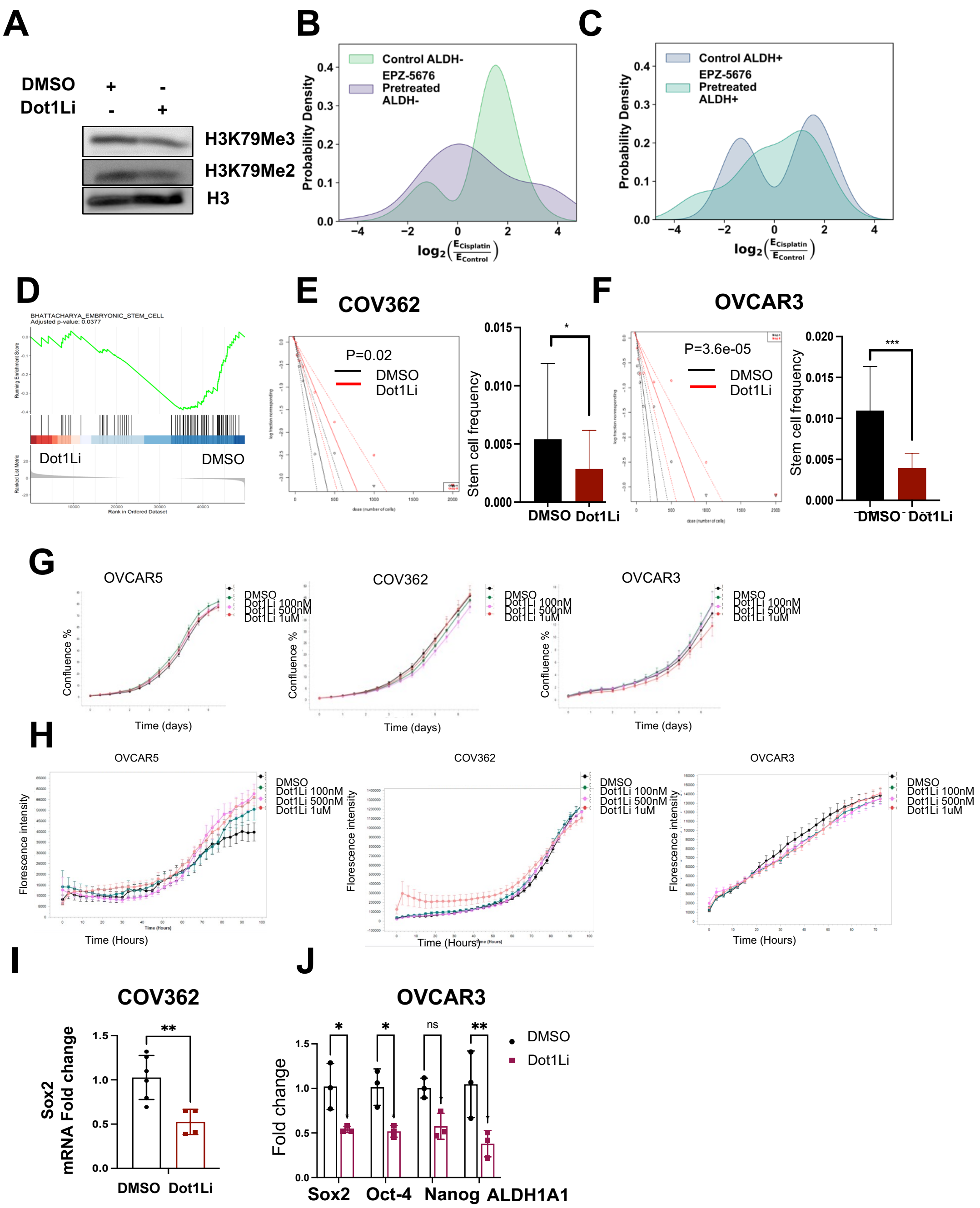

Supplemental Figure S8. Inhibition of Dot1L targets ovarian CSCs. (A) Western blot measured H3K79Me3, H3K79Me2, and histone H3 (loading control) protein levels in OVCAR5-derived ALDH+ CSCs treated with DMSO and Dot1L inhibitor (EPZ5676, 100nM) for 5 days (n=2). **(B-C)** Kernel density estimation (KDE) probability distribution functions (PDFs) of the differential gene expression induced by cisplatin on underexpressed genes. The magnitude of cisplatin-induced change in expression for underexpressed genes in control (DMSO treated) or Dot1Li treated **(B)** ALDH- cells and **(C)** ALDH+ cells. **(D)** GSEA analysis of DEGs in OVCAR5-ALDH+ cells treated with DMSO vs. Dot1Li was enriched in BHATTACHARYA_Embryonic_Stem_Cell pathway (FDR=0.0377). **(E-F)** Serial limited dilution number of **(E)** COV362 and **(F)** OVCAR3

cells (10, 25, 50, 100, 250, 500, 1000, 2000/well), were seeded into low-attached 96 well-plate and cultured in MammoCult medium (with DMSO or 100nM Dot1L inhibitor) for 7 days (n=12). The CSC frequency in the DMSO or Dor1Li (100nM, 5-days) was calculated by ELDA software and shown in the bar graph. **(G)** Incucyte analysis of time-lapse of cell confluency of OVCAR5, COV362 and OVCAR3 OC cells treated with DMSO or Dot1Li (100nM, 500nM, 1μM) for 7 days. **(H)** Incucyte analysis of time-lapse of Annexin V florescence intensity to determine cell apoptosis in OVCAR5, OVCAR3, COV362 cells treated with DMSO or Dot1Li (100nM, 500nM, 1μM) for 72 hours. **(I)** *mRNA* expression levels of *SOX2* in COV362 cells treated with DMSO or DOT1Li (1μM) for 5 days. Data are shown as means ± SD of biological replicates. **(J)** mRNA expression of stemness associated TFs *SOX2, OCT4, NANOG* and stemness gene *ALDH1A1* (n=3) in OVCAR3 cells treated with DMSO or DOT1Li (1μM) for 5 days **(***, *P* <0.05; **, *P*< 0.01; ***, *P*< 0.005).

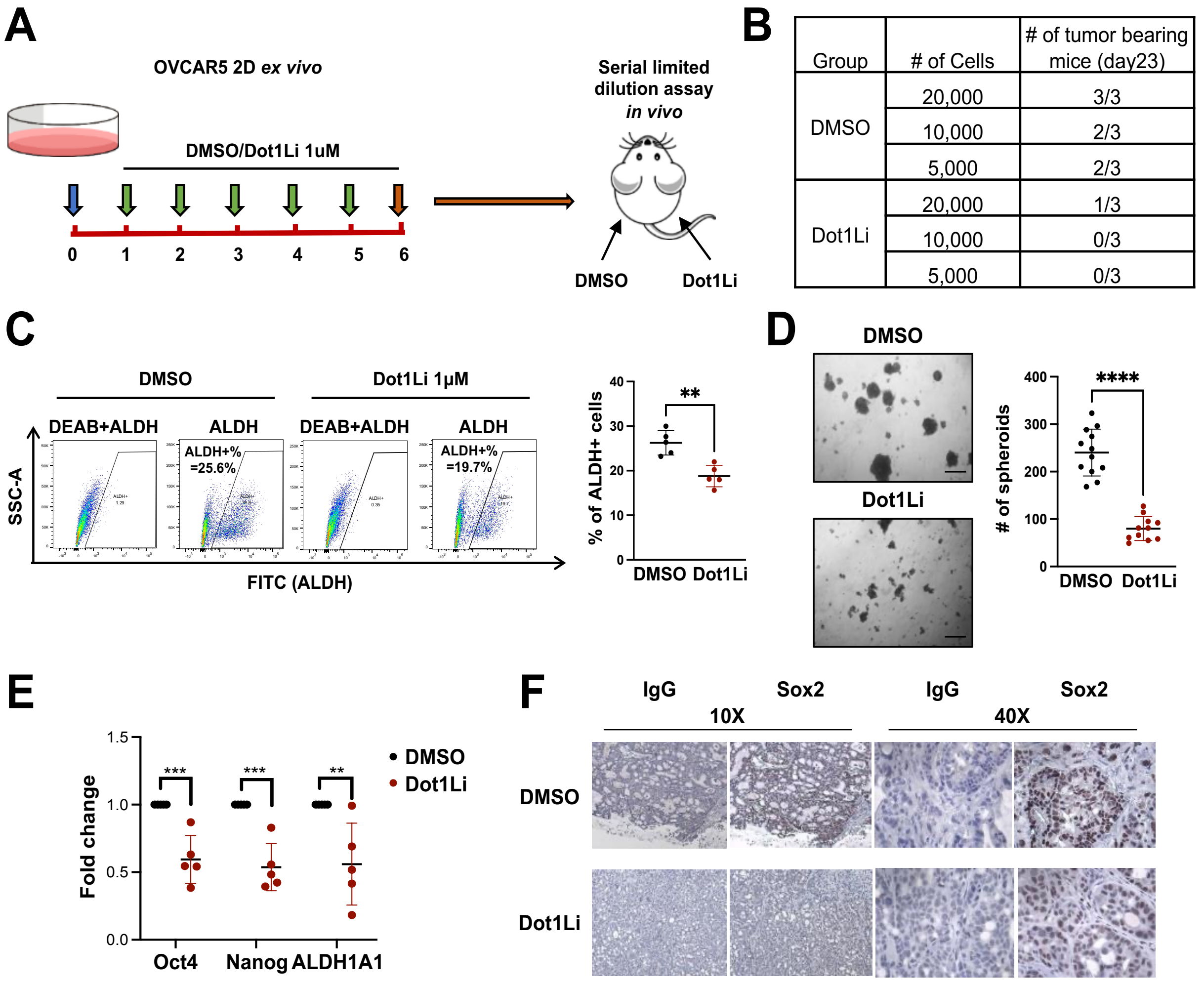

Supplemental Figure S9. Inhibition of Dot1L impedes tumor initiation by reducing CSCs. **(A)** The schematic indicates schedule of treatments of OVCAR5 cells ex vivo treated with DMSO or DOT1Li (1µM) for 5 days prior to SQ inoculation in nude mice. For tumor initiation experiments, serially diluted DMSO or DOT1Li (1µM) treated OVCAR5 cells (500, 1,000, and 2,500 cells) were SQ injected into nude mice. Tumor initiation and tumor growth were monitored. Tumors were harvested on Day 30 after cell injection. **(B)** The number of mice that developed SQ xenograft tumors in each group by Day 23 are shown. **(C)** ALDH+ CSCs population from xenografts collected from (**A**, n=4/group). **(D) Quantification of** spheroids formed by cells derived from xenografts collected from (**A**, n=4/group) measured by the CellTiter Glo 3D viability assay. Cells were isolated from xenografts from **A,** counted, and cultured in non-adherent plates and Mammocult media. Equal numbers of cells (5,000 cells/well) were plated for each group of xenografts (n=3/group). **(E)** *mRNA* expression levels of *OCT4*, *NANOG*, and *ALDH1A1* in cells derived from xenografts collected from (**A**, n=4/group). Data are shown as means ± SD of biological replicates. *, *P* <0.05; **, *P*< 0.01; ***, *P*< 0.005; and ****, *P*< 0.001). **(F)** Comparison of protein levels of *SOX2* measured by IHC in sections of xenografts collected from (**A**, n=4). IgG was used as negative control on consecutive section of the same tumor (similar area was photographed).

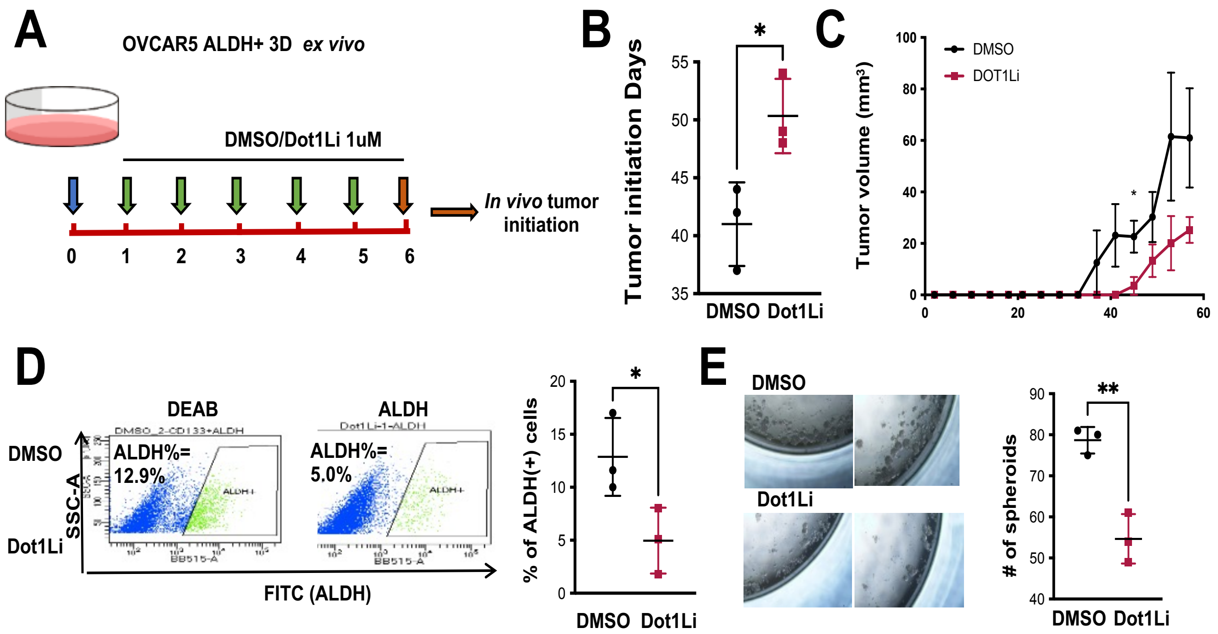

Supplemental Figure S10. Inhibition of Dot1L impedes tumor initiation *in vivo* by reducing CSCs. (A) The schematic indicates schedule of treatments of ALDH+ cells derived from OVCAR5 cells ex vivo treated with DMSO or DOT1Li (1µM) for 5 days prior to SQ inoculation in nude mice (n=3/group). Tumor initiation and tumor growth were monitored. **(B)** Time to tumor initiation and **(C)** tumor growth curve over time for tumors derived from ALDH+ cells treated with DMSO or DOT1Li (1 µM). **(D)** ALDH+ CSCs population from xenografts collected from (**A**, n=3/group). **(E)** Quantification of spheroids formed by cells derived from xenografts collected from (**A**, n=3/group) measured by the CellTiter Glo 3D viability assay. Data are shown as means ± SD of biological replicates. *, *P* <0.05; **, *P*< 0.01; ***, *P*< 0.005; and ****, *P*< 0.001).

References:

1. Zhang Y, Wang Y, Zhao G, Tanner EJ, Adli M, and Matei D. FOXK2 promotes ovarian cancer stemness by regulating the unfolded protein response pathway. 2022;132(10).
2. Wang Y, Calvert AE, Cardenas H, Rink JS, Nahotko D, Qiang W, et al. Nanoparticle Targeting in Chemo-Resistant Ovarian Cancer Reveals Dual Axis of Therapeutic Vulnerability Involving Cholesterol Uptake and Cell Redox Balance. *Adv Sci (Weinh).* 2024:e2305212.
3. Satpathy M, Cao L, Pincheira R, Emerson R, Bigsby R, Nakshatri H, et al. Enhanced peritoneal ovarian tumor dissemination by tissue transglutaminase. *Cancer Res.* 2007;67(15):7194-202.
4. Martin M. Cutadapt Removes Adapter Sequences From High-Throughput Sequencing Reads. *EMBnetjournal.* 2011;17(1):3.
5. Dobin A, Davis CA, Schlesinger F, Drenkow J, Zaleski C, Jha S, et al. STAR: ultrafast universal RNA-seq aligner. *Bioinformatics.* 2013;29(1):15-21.
6. Putri GH, Anders S, Pyl PT, Pimanda JE, and Zanini F. Analysing high-throughput sequencing data in Python with HTSeq 2.0. *Bioinformatics.* 2022;38(10):2943-5.
7. Chen Y, Chen L, Lun ATL, Baldoni PL, and Smyth GK. edgeR 4.0: powerful differential analysis of sequencing data with expanded functionality and improved support for small counts and larger datasets. *bioRxiv.* 2024:2024.01.21.576131.
8. Lee S, Lee S, Ouellette S, Park W-Y, Lee EA, and Park PJ. NGSCheckMate: software for validating sample identity in next-generation sequencing studies within and across data types. *Nucleic Acids Research.* 2017;45(11):e103-e.
9. Barretina J, Caponigro G, Stransky N, Venkatesan K, Margolin AA, Kim S, et al. The Cancer Cell Line Encyclopedia enables predictive modelling of anticancer drug sensitivity. *Nature.* 2012;483(7391):603-7.
10. clusterProfiler: an R Package for Comparing Biological Themes Among Gene Clusters. *OMICS: A Journal of Integrative Biology.* 2012;16(5):284-7.
11. Wu T, Hu E, Xu S, Chen M, Guo P, Dai Z, et al. clusterProfiler 4.0: A universal enrichment tool for interpreting omics data. *Innovation (Camb).* 2021;2(3):100141.
12. Mootha VK, Lindgren CM, Eriksson K-F, Subramanian A, Sihag S, Lehar J, et al. PGC-1α-responsive genes involved in oxidative phosphorylation are coordinately downregulated in human diabetes. *Nature Genetics.* 2003;34(3):267-73.
13. Subramanian A, Tamayo P, Mootha VK, Mukherjee S, Ebert BL, Gillette MA, et al. Gene set enrichment analysis: a knowledge-based approach for interpreting genome-wide expression profiles. *Proc Natl Acad Sci U S A.* 2005;102(43):15545-50.
14. Chen EY, Tan CM, Kou Y, Duan Q, Wang Z, Meirelles GV, et al. Enrichr: interactive and collaborative HTML5 gene list enrichment analysis tool. *BMC Bioinformatics.* 2013;14(1):128.
15. Kuleshov MV, Jones MR, Rouillard AD, Fernandez NF, Duan Q, Wang Z, et al. Enrichr: a comprehensive gene set enrichment analysis web server 2016 update. *Nucleic Acids Research.* 2016;44(W1):W90-W7.
16. Xie Z, Bailey A, Kuleshov MV, Clarke DJB, Evangelista JE, Jenkins SL, et al. Gene Set Knowledge Discovery with Enrichr. *Current Protocols.* 2021;1(3):e90.
